# Supplementary material for: Enhanced γ-globin reactivation and sickle cell correction through a repressor-to-activator motif switch in the HBG1/2 promoters
Source: Mol Ther Nucleic Acids. 2026 Jun 6;37(3):102974. doi: 10.1016/j.omtn.2026.102974 (PMC13330520; doi:10.1016/j.omtn.2026.102974)
Supplement: Document S1. Figures S1–S4 and Tables S2, S3, S5, and S6 [file mmc1.pdf]

OMTN, Volume *37*

Supplemental information

Enhanced γ-globin reactivation and sickle cell correction through a repressor-to-activator motif switch in the *HBG1/2* promoters

Anne Chalumeau, Panagiotis Antoniou, Maria Bou Dames, Pierre Martinucci, Elena Retana, Pragya Gupta, Mike Firth, Muralidhar Reddivari, Aathira Sarath Chandran, Jonathan S. Yen, Martin Peterka, Marcello Maresca, Mégane Brusson, and Annarita Miccio

Table S1: List of pegRNAs and springRNAs sequences used in the study (Provided separately in Excel format)

Table S2: List of ssODNs sequences targeting the -115 region of the *HBG* promoters

**ssODNs name**	**spacer 5'-3'**
sg115_D1(+)	CCCTGGCTAAACTCCACCCATGGGTTGGCCAGCCTTGCCTCTGCTGGGGACCCGATAACCAATAGCCTTGACAAGGCAAACTTGACCAATAGTCTTA
sg115_D1 (-)	TAAGACTATTGGTCAAGTTTGCCTTGTCAAGGCTATTGGTTATCGGGTCCCCAGCAGAGGCAAGGCTGGCCAACCCATGGGTGGAGTTTAGCCAGGG
sg115_D2 (+)	CTGAAACGGTCCCTGGCTAAACTCCACCCATGGGTTGGCCAGCCTTGCCTCTGCTGGGGACCCGATAACCAATAGCCTTGACAAGGCAAACTTGACCAATAGTCTTAGAGTATCCAG
sg115_D2 (-)	CTGGATACTCTAAGACTATTGGTCAAGTTTGCCTTGTCAAGGCTATTGGTTATCGGGTCCCCAGCAGAGGCAAGGCTGGCCAACCCATGGGTGGAGTTTAGCCAGGGACCGTTTCAG
sg115_D3 (+)	AAATATCTGTCTGAAACGGTCCCTGGCTAAACTCCACCCATGGGTTGGCCAGCCTTGCCTCTGCTGGGGACCCGATAACCAATAGCCTTGACAAGGCAAACTTGACCAATAGTCTTAGAGTATCCAGTGAGGCCAGG
sg115_D3 (-)	CCTGGCCTCACTGGATACTCTAAGACTATTGGTCAAGTTTGCCTTGTCAAGGCTATTGGTTATCGGGTCCCCAGCAGAGGCAAGGCTGGCCAACCCATGGGTGGAGTTTAGCCAGGGACCGTTTCAGACAGATATTT
sg115_D4 (+)	AAATATCTGTCTGAAACGGTCCCTGGCTAAACTCCACCCATGGGTTGGCCAGCCTTGCCTCTGCTGGGGACCCGATAACCAATAGCCTTGACAAGGCAAACTTGACCAATAGTCTTAGAGTATCCAGTGAGGCCAGGGGCCGGCGGCTGGCTAGGGATGAAGAATAAAAGGAAGCACCCTTCAGCAG
sg115_D4 (-)	CTGCTGAAGGGTGCTTCCTTTTATTCTTCATCCCTAGCCAGCCGCCGGCCCCTGGCCTCACTGGATACTCTAAGACTATTGGTCAAGTTTGCCTTGTCAAGGCTATTGGTTATCGGGTCCCCAGCAGAGGCAAGGCTGGCCAACCCATGGGTGGAGTTTAGCCAGGGACCGT
sg115_D5 (+)	TTAAGCAGCAGTATCCTCTTGGGGGCCCCTTCCCCACACTATCTCAATGCAAATATCTGTCTGAAACGGTCCCTGGCTAAACTCCACCCATGGGTTGGCCAGCCTTGCCTCTGCTGGGGACCCGATAACCAATAGCCTTGACAAGGCAAACTTGACCAATAGTCTTAGAGTATCCAGTGAGGCCAGG
sg115_D5 (-)	CCTGGCCTCACTGGATACTCTAAGACTATTGGTCAAGTTTGCCTTGTCAAGGCTATTGGTTATCGGGTCCCCAGCAGAGGCAAGGCTGGCCAACCCATGGGTGGAGTTTAGCCAGGGACCGTTTCAGACAGATATTTGCATTGAGATAGTGTGGGGAAGGGGCCCCCAAGAG

ssODNs are labeled numerically (1–5) to denote distinct designs, with (+) and (–) indicating the DNA strand targeted by the ssODN.

Table S3: List of primers used for on- and off-target analysis by deep sequencing

**Deep sequencing for PEn experiments**				
**Analysis**	**Amplified region**	**F/R**	**N.**	**Sequence (5' to 3')**
On-target	*chr11_5271202/5276126*	F	1	TCGTCGGCAGCGTCAGATGTGTATAAGAGACAGAAGCGGAATGACTGAATCGGAACAAGG
			2	TCGTCGGCAGCGTCAGATGTGTATAAGAGACAGCGTTGGAATGACTGAATCGGAACAAGG
			3	TCGTCGGCAGCGTCAGATGTGTATAAGAGACAGGTCAGGAATGACTGAATCGGAACAAGG
			4	TCGTCGGCAGCGTCAGATGTGTATAAGAGACAGACAGGGAATGACTGAATCGGAACAAGG
			5	TCGTCGGCAGCGTCAGATGTGTATAAGAGACAGCAGTGGAATGACTGAATCGGAACAAGG
			6	TCGTCGGCAGCGTCAGATGTGTATAAGAGACAGTGTCGGAATGACTGAATCGGAACAAGG
		R	1	GTCTCGTGGGCTCGGAGATGTGTATAAGAGACAGAGCAGCACTGGCCTCACTGGATACTCT
			2	GTCTCGTGGGCTCGGAGATGTGTATAAGAGACAGCCTTGCACTGGCCTCACTGGATACTCT
			3	GTCTCGTGGGCTCGGAGATGTGTATAAGAGACAGGAACGCACTGGCCTCACTGGATACTCT
			4	GTCTCGTGGGCTCGGAGATGTGTATAAGAGACAGTTGGGCACTGGCCTCACTGGATACTCT
			5	GTCTCGTGGGCTCGGAGATGTGTATAAGAGACAGACAGGCACTGGCCTCACTGGATACTCT
			6	GTCTCGTGGGCTCGGAGATGTGTATAAGAGACAGCAGTGCACTGGCCTCACTGGATACTCT
			7	GTCTCGTGGGCTCGGAGATGTGTATAAGAGACAGCTACGCACTGGCCTCACTGGATACTCT
			8	GTCTCGTGGGCTCGGAGATGTGTATAAGAGACAGGATGGCACTGGCCTCACTGGATACTCT
			9	GTCTCGTGGGCTCGGAGATGTGTATAAGAGACAGGGATGCACTGGCCTCACTGGATACTCT
			10	GTCTCGTGGGCTCGGAGATGTGTATAAGAGACAGTGTCGCACTGGCCTCACTGGATACTCT
Off-target	*chr2_176540234*	F	1	ATTGGGGGAGACAGACCCATG
		R	2	CATTTTTATCCAGCTTGGCAGGGG

F, forward primers; R, reverse primers

Table S4: Crispresso2 analysis (Provided separately in Excel format)

Table S5: List of primers used for RT-qPCR analysis

Amplified region	F/R	Sequence (5' to 3')
HBA	F	CGGTCAACTTCAAGCTCCTAA
	R	ACAGAAGCCAGGAACTTGTC
HBB	F	GCAAGGTGAACGTGGATGAAGT
	R	TAACAGCATCAGGAGTGGACAGA
HBG1 + HBG2	F	CCTGTCCTCTGCCTCTGCC
	R	GGATTGCCAAAACGGTCAC
CDKN1A	F	CAGCATGACAGATTTCTACCACTC
	R	CTCGCGCTTCCAGGACTG
GAPDH	F	ACCACAGTCCATGCCATCACT
	R	CCATCACGCCACAGTTTCC

Table S6: Primers and probe used for the 4.9-kb deletion ddPCR analysis

ddPCR analysis			
	F/R	Sequence (5' to 3')	Fluorophore
primers	F	AAAGAGAGGTGGAAATGAGG	
	R	CCACTTTGACTGAGCCAATA	
HBG1	probe	CCAGTAGAAAGAACTTTCATCTTTCCCTC	FAM
HBG2	probe	CTTTCCCCTATTTTTGTTATTCGTTTTA	VIC

Figure S1

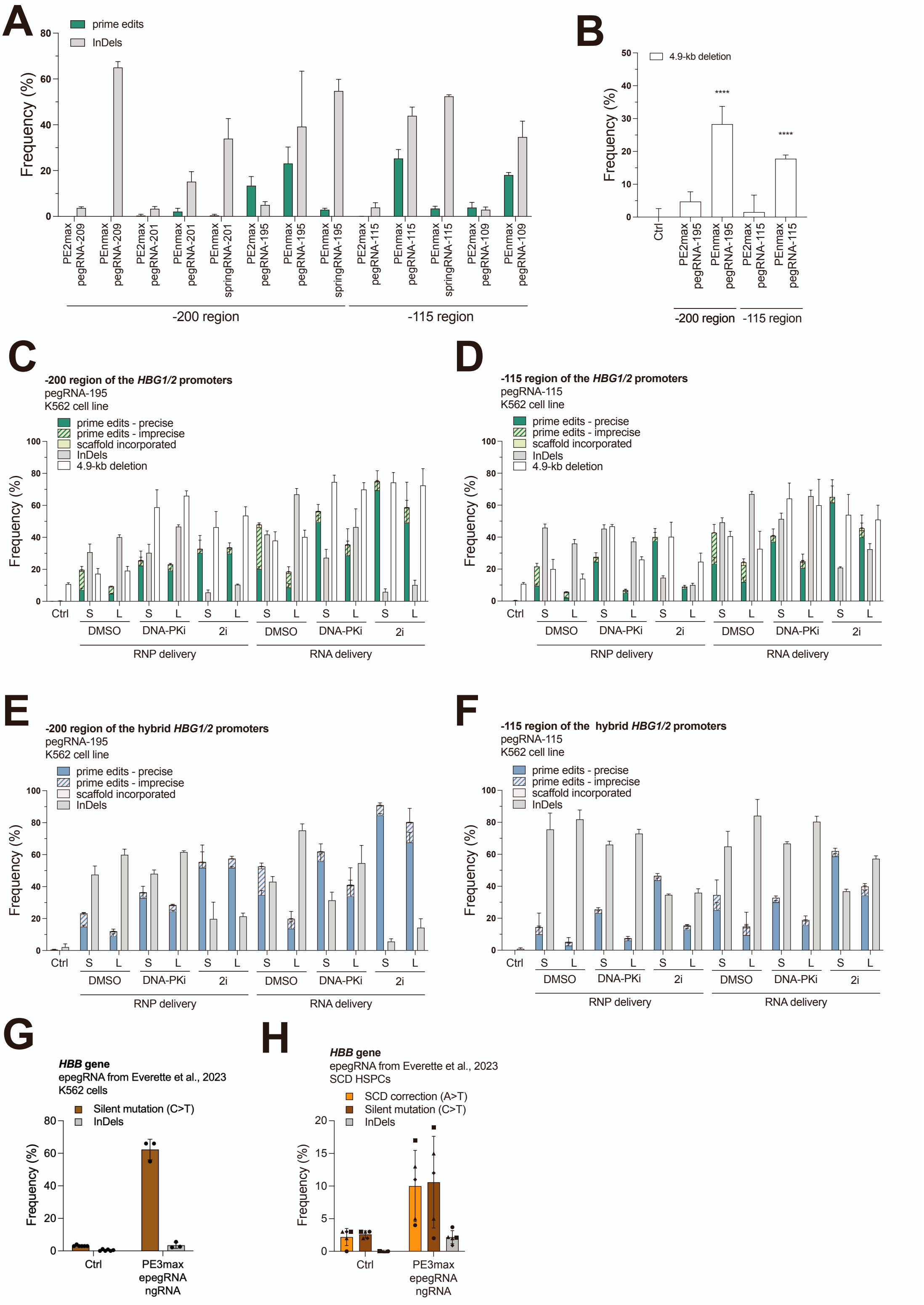

Figure S1: Design and screening of pegRNAs and springRNAs targeting the *HBG1/2* promoters in K562 cells.

(A) Frequency of the desired insertion (prime edits) and InDels in the *HBG1/2* promoters induced by pegRNAs or the springRNAs in combination with PEnmax or PE2max in K562 cells. The *HBG1/2* promoters were amplified and subjected to Sanger sequencing. Data were analyzed using the TIDE software. Bars represent the mean ± SD of 3 biologically independent replicates.
(B) The frequency of the 4.9-kb deletion was measured by ddPCR in control (Ctrl, cells transfected with Tris-EDTA, TE) and edited cells.
(C, D) Percentage of total *HBG1/2* promoters containing the total prime edits (precise, imprecise, scaffold incorporated) and the InDels induced by (C) pegRNA-195_S or pegRNA-115_L, or (D) pegRNA-115_S or pegRNA-115_L, delivered as RNA or RNP in K562 cells treated or not with DNA-PKi or DNA-PKi and Polθi inhibitors. The *HBG1/2* promoters were amplified and subjected to NGS. Data were analyzed with the CRISPResso2 webtool. We report also the frequency of the 4.9-kb deletion measured by ddPCR for control (ctrl, TE-transfected cells) and prime-edited conditions. Bars represent the mean ± SD of 3 technical replicates.
(E, F) Percentage of the hybrid *HBG1/2* promoters containing the prime edits (precise, imprecise, scaffold incorporated) and InDels induced by (E) pegRNA-195_S or pegRNA-115_L or (F) pegRNA-115_S or pegRNA-115_L, delivered as RNA or RNP in K562 cells treated or not with DNA-PKi or DNA-PKi and Polθi inhibitors. The hybrid *HBG1/2* promoters were amplified and subjected to NGS. Data were analyzed with the CRISPResso2 webtool. Bars represent the mean ± SD of 3 biological replicates.
(G) Frequency of insertion of the silent mutation in the *HBB* gene, and InDels in the *HBB* gene induced by epegRNA PE3max in K562 cells (n=3). The *HBB* gene was amplified and subjected to Sanger sequencing. Data were analyzed using the TIDE software. Bars represent the mean ± SD of 3 biologically independent replicates.
(H) Frequency of SCD mutation correction and insertion of a silent mutation, and InDels in the *HBB* gene induced by epegRNA PE3max in HSPCs from patients with SCD (n=4 donors). The *HBB* gene was amplified and subjected to Sanger sequencing. Data were analyzed using the TIDE software. Bars represent the mean ± SD of 5 biologically independent replicates.

Figure S2

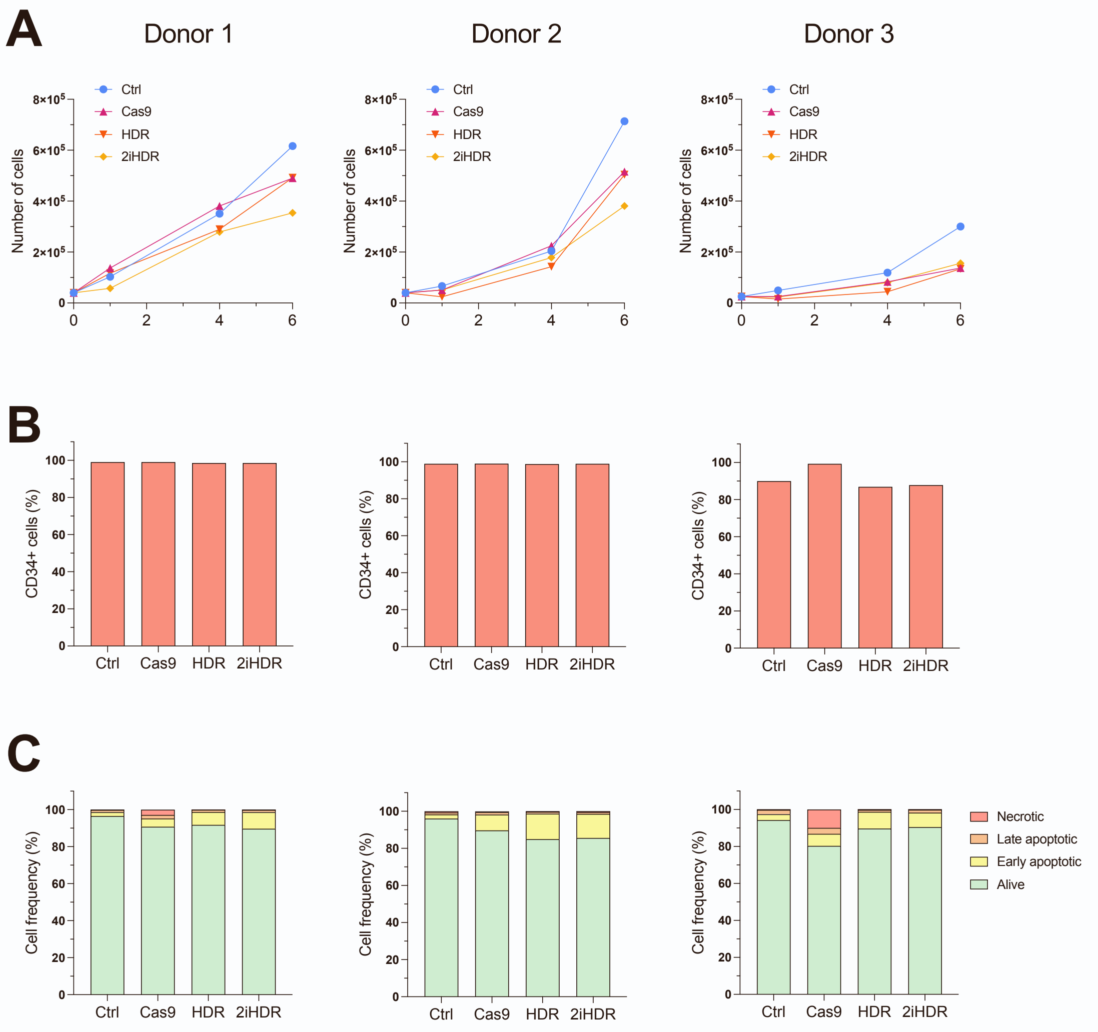

Figure S2: Cell recovery, viability, and DNA damage response in control and edited HSPCs

(A) Number of HSPCs 1, 2, 4 and 6 days post transfection in control (TE-transfected) and edited samples.

(B) Frequency of CD34+ HSPCs 24 h post transfection in control (TE-transfected) and edited samples, as measured by flow cytometry.

(C) Frequency of alive, early apoptotic, late apoptotic, and necrotic HSPCs 24 h post transfection, as measured by flow cytometry after annexin-V staining in control (mock transfected) and edited samples.

(A-C) Data are shown for 3 different SCD donors, including one bone marrow sample (donor 3).

Figure S3

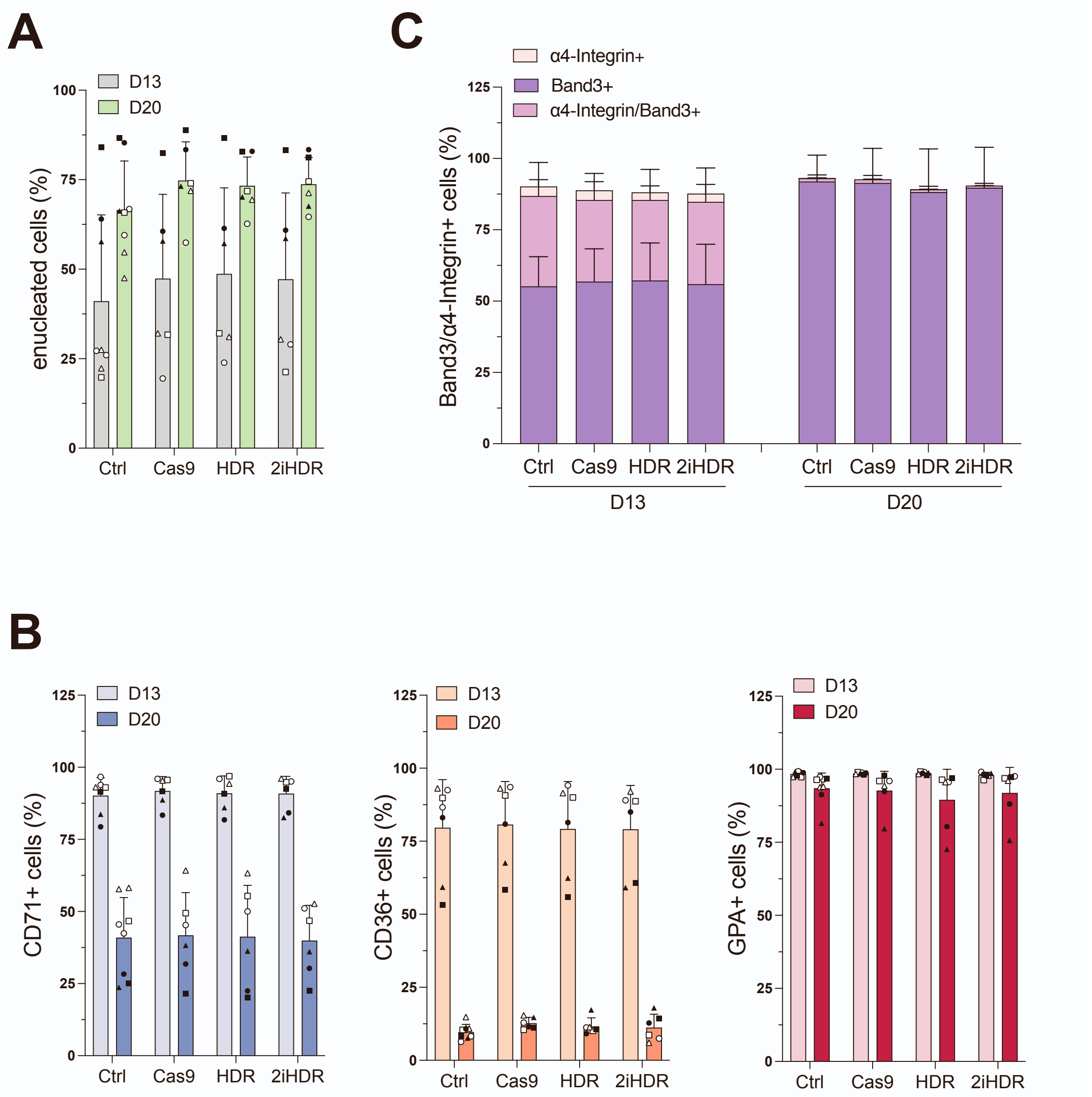

Figure S3: The Cas9-based strategies do not impact the erythroid differentiation
(A) Frequency of enucleated cells at day 13 and 20 of erythroid differentiation, as measured by flow cytometry analysis after DRAQ5 nuclear staining in control and edited samples. Data are expressed as mean ± SD (n=6 biologically independent experiments; 5 different donors). No statistical differences were observed between control and edited samples using two-way ANOVA with Tukey's multiple comparisons test.
(B) Frequency of (left) CD71+, (middle) CD36+ and (right) GPA+ cells at day 13 and 20 of the erythroid differentiation, as measured by flow cytometry analysis of CD36, CD71, and GPA erythroid markers in control and edited samples. Data are expressed as mean ± SD (n=6 biologically independent experiments; 5 different donors). No statistical differences were observed between control and edited samples using two-way ANOVA with Tukey's multiple comparisons test.

(C) Frequency of α4-Integrin+, Band3+ and α4-Integrin+/Band3+ in 7AAD-/GPA+ cells at day 13 and 20 of erythroid differentiation, as measured by flow cytometry analysis of α4-Integrin and Band3 erythroid markers in control and edited samples. Data are expressed as mean ± SD (n=6 biologically independent experiments; 5 different donors). No statistical differences were observed between control and edited samples using two-way ANOVA with Tukey's multiple comparisons test.

Figures S4

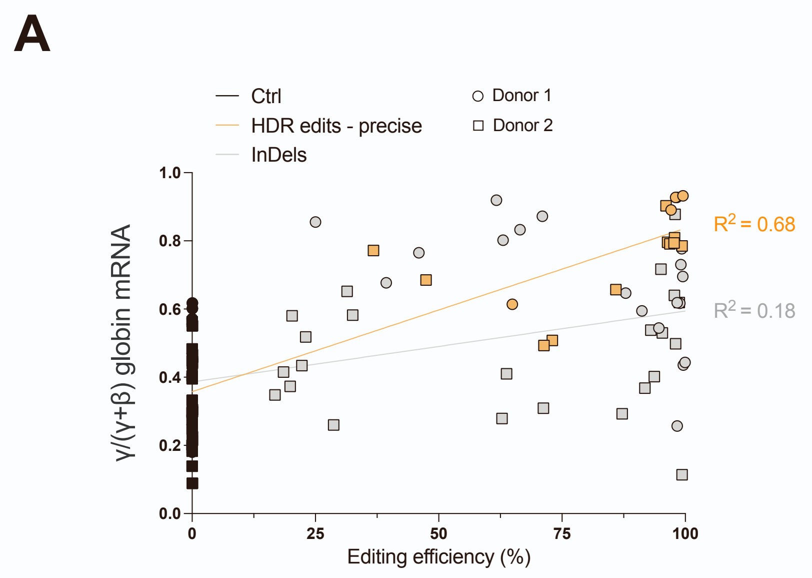

Figure S4: Cas9-HDR-edited BFU-E express high HbF levels.
(A) Correlation between γ-globin mRNA expression and editing efficiency in single erythroid BFU-E (n=32 to 74 single BFU-E for each group). γ-globin mRNA expression was normalized to α-globin mRNA and expressed as a percentage of the total β- and γ- globin mRNA. For BFU-E containing the HDR precise edits, R^2 = 0.68, Y = 0,004792*X + 0,3577; for BFU-E containing InDels, R^2 = 0.18, Y = 0,002081*X + 0,3862 (simple linear regression).
