# Supplementary material for: Enhanced γ-globin reactivation and sickle cell correction through a repressor-to-activator motif switch in the HBG1/2 promoters
Source: Mol Ther Nucleic Acids. 2026 Jun 6;37(3):102974. doi: 10.1016/j.omtn.2026.102974 (PMC13330520; doi:10.1016/j.omtn.2026.102974)
Supplement: Document S3. Article plus supplemental information [file mmc3.pdf]

Molecular Therapy
Nucleic Acids
Original Article

Enhanced γ-globin reactivation and sickle cell correction through a repressor-to-activator motif switch in the *HBG1/2* promoters

Anne Chalumeau,[1] Panagiotis Antoniou,[2] Maria Bou Dames,[1] Pierre Martinucci,[1] Elena Retana,[1] Pragya Gupta,[2] Mike Firth,[2] Muralidhar Reddivari,[3] Aathira Sarath Chandran,[3] Jonathan S. Yen,[3] Martin Peterka,[2] Marcello Maresca,[2,4] Mégane Brusson,[1,4] and Annarita Miccio[1,4]

[1]Université Paris Cité, Imagine Institute, Laboratory of Chromatin and Gene regulation During Development, INSERM UMR1163, 75015 Paris, France; [2]Genome Engineering, Discovery Sciences, R&D, AstraZeneca, Gothenburg, Sweden; [3]Department of Hematology, St. Jude Children's Research Hospital, Memphis, TN, USA

Sickle cell disease (SCD) is caused by the production of an abnormal adult hemoglobin that generates sickle-shaped red blood cells (RBCs). Transplantation of autologous genetically corrected hematopoietic stem/progenitor cells (HSPCs) represents a promising therapy. Persistent fetal hemoglobin expression improves SCD. Here, we engineered the fetal *HBG1/2* promoters by replacing the BCL11A repressor-binding site (BS) with a TAL1:GATA1 motif recognized by transcriptional activators. We exploited the prime editing nuclease (PEn) that efficiently installed the TAL1:GATA1 motif in K562 cells, outperforming the original PE. Non-homologous end joining (NHEJ) and/or alternative-end joining (alt-EJ) pathway inhibition enhanced precise editing. However, this strategy was poorly efficient in patients' HSPCs. Alternatively, we used CRISPR-Cas9 nuclease to either disrupt the BCL11A BS via NHEJ and/or alt-EJ or to replace it with the TAL1:GATA1 motif via homology-directed repair (HDR) using a donor ssODN template. NHEJ and alt-EJ inhibition improved product purity, reducing insertions and deletions (indels), and achieving superior precise editing efficiency compared to PEn in K562 and HSPCs. HDR-edited HSPCs preserved clonogenic capacity and differentiated into RBCs showing elevated *HBG* expression and correction of the sickling phenotype. These results indicate that replacing the BCL11A BS with a TAL1:GATA1 motif is a potent strategy for reactivating *HBG1/2* to treat SCD.

INTRODUCTION

Beta-hemoglobinopathies are genetic disorders caused by mutations that either reduce adult β-globin chain production (β-thalassemia) or generate an abnormal β-globin chain (sickle cell disease, SCD). Beta-thalassemia and SCD are the most common inherited genetic disorders affecting millions of people worldwide. In β-thalassemia, reduced β-globin synthesis leads to inadequate hemoglobinization of red blood cells (RBCs) and anemia.[1] In SCD, a single point mutation in the β-globin (*HBB*) gene generates the sickle β^S-globin chain and hemoglobin S (HbS) that polymerizes under hypoxic conditions.[2] HbS polymerization triggers RBC sickling, ultimately resulting in vaso-occlusive crises, hemolytic anemia, and organ damage. Current symptomatic treatments, such as RBC transfusions and supportive care, are associated with high costs and continue to result in a poor quality of life.[2,3] A curative option is allogeneic transplantation of hematopoietic stem and progenitors cells (HSPCs), but this is limited by immunological risks and availability of compatible donors.[4]

The clinical severity of β-hemoglobinopathies is alleviated by the co-inheritance of genetic mutations termed hereditary persistence of fetal hemoglobin (HPFH), which promote continued expression of the fetal γ-globin and production of fetal hemoglobin (HbF) in adult life.[5] Gamma-globin compensates for β chain deficiency in β-thalassemia and exerts an anti-sickling effect in SCD. Naturally occurring HPFH mutations identified in the promoters of the two γ-globin genes (*HBG1/2)* are known to either generate *de novo* DNA motifs recognized by potent transcriptional activators (e.g., KLF1,[6] TAL1,[7] and GATA1[8]) or disrupt/delete transcriptional repressor (e.g., LRF[9] and BCL11A[9]) binding sites (BSs). Interestingly, the co-occurrence of multiple HPFH mutations is associated with higher HbF levels compared to individual mutations.[10]

Several genome editing strategies have been developed to genetically modify autologous HSPCs and treat patients with β-hemoglobinopathies. Most of these approaches are based on the use of a single-guide RNA (sgRNA) that drives the *SpCas9* (Cas9) nuclease to the target sequence where it generates a double-strand

Received 8 October 2025; accepted 4 June 2026;
https://doi.org/10.1016/j.omtn.2026.102974.

[4]These authors contributed equally

Correspondence: Mégane Brusson, Imagine Institute, 24, Boulevard du Montparnasse, 75015 Paris, France.
E-mail: megane.brusson@institutimagine.org
Correspondence: Annarita Miccio, Imagine Institute, 24, Boulevard du Montparnasse, 75015 Paris, France.
E-mail: annarita.miccio@institutimagine.org

Published by Elsevier Inc. on behalf of The American Society of Gene and Cell Therapy.
This is an open access article under the CC BY license (http://creativecommons.org/licenses/by/4.0/).

break (DSB). The DSB is then mainly repaired via the non-homologous end joining (NHEJ)[11] or alternative-end joining (alt-EJ, also called micro-homology-mediated end joining, MMEJ) pathways[12,13] leading to the generation of small insertions and deletions (indels). This can be exploited to knockout genes or inactivate regulatory elements. CRISPR-Cas9 has been used to inactivate the erythroid enhancer of *BCL11A*, a gene encoding a major HbF repressor,[14,15] showing promising results in pre-clinical and clinical trials. This CRISPR-Cas9 therapy (exagamglogene autotemcel) was approved by the FDA to treat patients with β-hemoglobinopathies.[16,17] However, *BCL11A* knock-down can affect erythropoiesis, prompting the search for more suitable therapeutic targets.[18,19] CRISPR-Cas9 has also been used to reactivate HbF by disrupting the LRF repressor BS[20,21] or the BCL11A BS[20,22–24] in the −200 region and the −115 region of the *HBG1/2* promoters, respectively, via generation of indels (predominantly deletions) through NHEJ or alt-EJ. In particular, approximately 30%–50% of these deletions are associated with microhomology (MH) motifs at the target site, suggesting DNA repair via alt-EJ.[20] Precise genome editing can be achieved upon Cas9-mediated DSBs via homology-directed repair (HDR) in the presence of a DNA donor template. Cas9-HDR approaches are less efficient than NHEJ-based strategies in quiescent HSPCs; however, several optimizations have been carried out to increase Cas9-HDR efficiency, such as the use of NHEJ pathway inhibitors.[25,26]

Prime editing is a recently developed technology enabling all twelve possible base conversions, as well as targeted insertions, deletions, or combined edits[27] in a specific region without generating DSBs. Prime editing utilizes a prime editor (PE)—a Cas9 nickase (Cas9n) fused to an engineered reverse transcriptase (RT)—together with a prime editing guide RNA (pegRNA) containing both an RT-template (RTT) with the desired edits and a primer-binding site, which allows direct incorporation of edits into genomic DNA (PE2). To improve processivity and editing activity, the enzyme has been codon optimized and engineered by adding nuclear localization signal (NLS) sequences, linkers, and the R221K and N394K mutations in the Cas9n to improve its activity, generating the PEmax.[28,29] Despite all these improvements, some target regions remained hard-to-edit with the nickase-based PE, especially when DNA insertions are desired. We previously showed that this limitation can be overcome by replacing the nickase with a Cas9 nuclease, generating PEnmax, which supports precise insertion of DNA sequences and improves editing efficiency for pegRNAs that were inefficient with the nickase-based PE.[30] Of note, in the PEn-based strategy, a 30-nt long homology tail is added to the 5′ extremity of the RTT of the pegRNA to favor edit incorporation.

In this study, we applied different genome-editing tools to efficiently disrupt repressors BSs in the *HBG1/2* promoters and simultaneously insert a 18-bp long TAL1:GATA1 composite motif, which is recognized by two potent transcriptional activators TAL1 and GATA1[31,32] to upregulate HbF. We demonstrated that replacing the BCL11A BS with the TAL1:GATA1 motif in SCD HSPCs, increased HbF levels and corrected the sickling phenotype in the erythroid progeny, outperforming approaches that only disrupted the BCL11A BS. Notably, the Cas9-HDR-based strategies yielded a higher frequency of precise edits than prime-editing approaches.

RESULTS

PEn outperforms PE to replace repressor BSs with a TAL1:GATA1 motif in the *HBG1/2* promoters

We developed editing strategies that aim at replacing repressors BSs with a composite 18-bp-long TAL1:GATA1 motif recognized by two potent transcriptional activators (Figure 1A) to generate a modified γ-globin promoter with increased HbF production. We used the motif CRGCTGBNGNSNNSAGATAA that contains TAL1 and GATA1 BSs and is present in genes highly expressed during erythroid development.[31,32] We mapped this motif to the β-globin locus control region (LCR) and selected a sequence (CTGCTGGGGACCCAGATAA) in the DNAse I hypersensitive site 2 of the LCR. Finally, we slightly adapted the GATA1 BS by removing the first adenine (GATAA, instead of AGATAA) as this nucleotide is less critical for GATA1 binding.[33]

First, we designed several pegRNAs (targeting the LRF and the BCL11A BS in the −200 and −115 regions of the *HBG1/2* promoters, respectively) with spacer sequences used in previously developed sgRNAs, and pegRNAs showing high editing efficiency and low off-target activity in combination with Cas9 nuclease[20,34] (Figure 1B). All pegRNAs carry an RTT (with a length ranging from 48- to 55-nt) containing a total or partial deletion of the BS depending on the sgRNA spacer (inducing a cleavage site inside or outside the repressor BS, respectively), the 18-bp long TAL1:GATA1 sequence, and a 30-nt long homology tail (or 36-nt long for the pegRNA-195) (Figures 1A and 1B; Table S1). For pegRNA-201, pegRNA-195, and pegRNA-115, which induce a cleavage site inside the repressor BS, we also designed springRNA to exploit the primed insertions strategy (PRINS), which is known to promote insertions at the DSB through NHEJ.[30,35] The springRNAs contain the same spacer and PBS of the corresponding pegRNAs with an RTT carrying the 18-nt TAL1:GATA1 sequence that after reverse transcription is directly ligated at the DSB site by NHEJ (Figure 1B). This allows the insertion of the desired motif into the repressor BS, causing its inactivation.

We tested the different constructs by plasmid transfection in K562 cells comparing PE2max to the PEnmax (Figure 1C). We initially evaluated the editing efficiency by Sanger sequencing. Overall, PEnmax outperformed PE2max in editing both −115 and −200 sites (Figure S1A). As expected, PEn induced a higher frequency of indels compared to PE2max (Figure S1A). In addition, none of the three springRNAs outperformed PE2max or PEnmax at inserting the expected motif (Figure S1A).

To better characterize the DNA repair profile induced by the most efficient editing strategies, *HBG1/2* promoters were amplified by PCR and subjected to NGS (Figure 1D). The frequency of total prime-editing events (i.e., precise, imprecise, and edits incorporating the scaffold) approached 30% for pegRNA-195 and

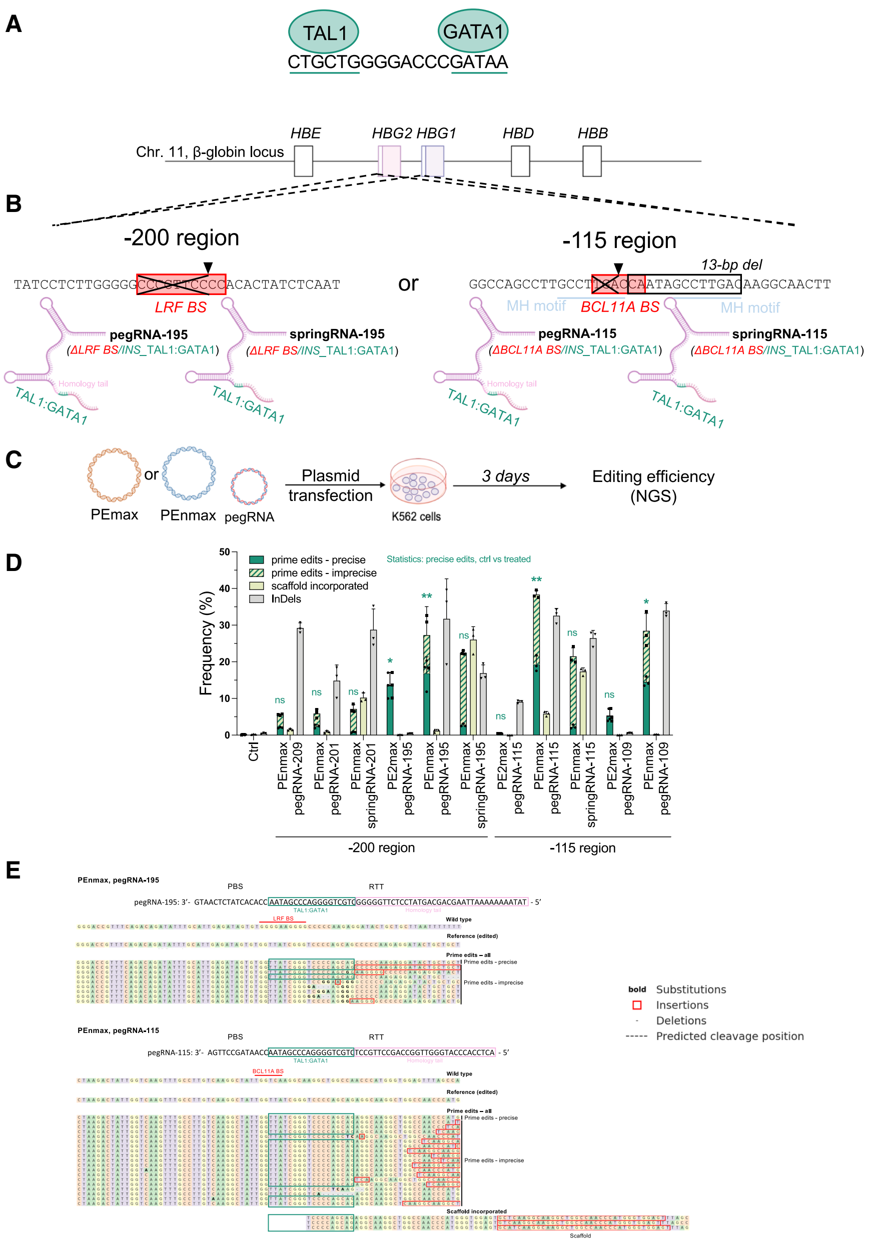

Figure 1. Design and screening of pegRNAs targeting the *HBG1/2* promoters in K562 cells

(A) Representation of the *in vitro* consensus sequence of the composite 18-nt long TAL1:GATA1 motif. (B) Schematic representation of the β-globin locus on chromosome (chr.) 11 including the β-like globin genes (*HBE*, *HBG1*, *HBG2*, *HBD*, and *HBB*) and the *HBG1/2* promoters. The LRF and BCL11A repressor-binding sites (BSs) are highlighted by a light red box. The pegRNA-195 and springRNA-195 and the pegRNA-115 and springRNA-115, targeting respectively the −200 and the −115 region of the *HBG1/2* promoters, allow the replacement of the LRF or the BCL11A repressor BSs (ΔBCL11A or ΔLRF BS) with the consensus TAL1:GATA1 sequence (INS_TAL1:GATA1). SpringRNAs do not contain the homology tail. The 13-bp HPFH deletion (13-bp del), indicated by an empty black box, occurs via alt-EJ after DSB because of the 8-bp microhomology motifs (MH motifs, blue lines) located in the −115 region.[20] Black arrowheads indicate the corresponding springRNA/pegRNA's cleavage sites at position −195 and −115 from the transcriptional start site (TSS) of the *HBG1/2* genes. (C) Experimental protocol used to test prime editing strategies in K562 cells. Plasmids expressing PEnmax or PE2max and a pegRNA or a springRNA were co-transfected in K562 cells. The prime editing efficiency is assessed by PCR amplification of the *HBG1/2* promoters and NGS 3 days after transfection. Data were analyzed with the CRISPResso2 webtool. Samples transfected with TE (Tris-EDTA) buffer were used as controls (Ctrl). (D) Percentage of NGS reads containing the prime-editing events (precise, imprecise, and scaffold incorporated) and indels. Bars represent the mean ± SD of 3 biological replicates. Statistical significance for "prime edits—precise" was assessed using a Kruskal-Wallis test followed by Dunn's multiple comparisons test, with the "Ctrl" condition used as the reference condition. **p* < 0.05, ***p* < 0.01. ns, not significant. (E) CRISPResso2 alignments of the *HBG1/2* promoter sequences, as determined by NGS, in samples edited with pegRNA-195 (upper) or pegRNA-115 (lower) in combination with PEnmax. We reported the PBS and RTT sequences of the pegRNAs, the wild type sequence (no prime editing event), the reference (i.e., the expected edited sequence used by CRISPResso2 to align NGS reads), all prime edits (including precise and imprecise events) and "scaffold incorporated" editing events (identified for pegRNA-115). The TAL1:GATA1 motif and the homology tail are highlighted with a green and a pink box, respectively. BCL11A and LRF BSs are indicated with a red box in the wild-type sequence.

pegRNA-109, and 45% for pegRNA-115 using the PEnmax, of which the precise edits represent around 17%, 15%, and 19% of total NGS reads, respectively (Figure 1D). As shown by Sanger sequencing, the PEnmax outperformed the PEmax in generating precise and imprecise edits (Figure 1D). PRINS (using springRNA and the PEnmax) generated mainly imprecise edits or edits containing the pegRNA scaffold, which was consistent with NHEJ-mediated capture of pegRNA-derived sequences during PRINS editing (Figure 1D). Among the three best-performing pegRNAs, we selected pegRNA-195 and pegRNA-115 for further analyses, as these pegRNAs generate a cleavage site inside the repressor BS, favoring its disruption, and are associated with the highest frequency of precise edits. The "imprecise" prime edits observed using pegRNA-195 and pegRNA-115 correspond mainly to the deletion of the repressor BSs and the introduction of a complete or partial TAL1:GATA1 motif with other substitutions, insertions (i.e., duplications of part of the RTT) or deletions (Figures 1D and 1E). The "scaffold incorporated" edits are characterized by integration of the TAL1:GATA1 motif, introduction of the RTT and partial incorporation of the scaffold sequence; these

modifications lead also the disruption of the repressor BSs (Figure 1E). Consequently, both imprecise and scaffold-incorporated edits are also expected to reactivate γ-globin expression similar to indels that inactivate the repressor BSs.[20] Furthermore, we observed that the PEn system generates a higher proportion of *HBG1/2* 4.9-kb deletions compared to PEmax-based strategies. This deletion event is the consequence of concurrent cleavage of the two identical *HBG1/2* promoter regions resulting in the loss of the *HBG2* gene, a phenomenon also observed in Cas9 nuclease- and base editor-mediated approaches[20,36] (Figure S1B).

The prime-editing efficiency is modest in primary SCD HSPCs despite the optimization of pegRNA and delivery method

Prior to evaluating PEn strategies in patients' cells, we further optimized the pegRNA-195 and the pegRNA-115 to facilitate their synthesis—specifically aligning with current protocols optimized for RNA with a maximum length of 150 nt- and to improve the editing efficiency. To meet this length criteria, we generated pegRNA-195_S and pegRNA-115_S, harboring a shorter (S) homology tail of 16- and 7-nt, respectively, and we compared them with the original design constructs (L; Figure 2A). We tested pegRNA-195_S and pegRNA-115_S with PEnmax in K562 cells using different delivery methods (i.e., ribonucleoprotein [RNP] or RNA transfection) that are suitable for clinical applications.[21,36] The editing efficiency was evaluated by NGS to quantify the frequency of desired insertion and alternative modifications (Figure 2A). The pegRNAs with the shortest homology tail inserted both precise and imprecise edits at higher efficiency compared to long pegRNAs, while the incorporation of the scaffold remained low (Figures 2B and 2C). Overall, the frequency of precise and imprecise edits was greater upon RNA transfection compared to RNP delivery and were significantly higher for both short pegRNA upon RNA transfection compared to control treated cells (Figures 2B and 2C). Importantly, the proportion of indels (likely less productive in HbF reactivation compared to precise and imprecise edits) was reduced using short pegRNAs upon RNA delivery (Figures 2B and 2C). The frequency of 4.9-kb deletion, measured by droplet digital PCR (ddPCR), was comparable between S and L constructs and higher in RNA-transfected samples (Figures 2B and 2C). These results suggest that RNA delivery, by increasing pegRNA/PEn concentration in the cells compared to RNP transfection, may facilitate the simultaneous cleavage of the two promoters (Figures 2B and 2C).

Subsequently, we characterized the editing profile of the hybrid *HBG1/2* promoters that are generated following the 4.9-kb deletion by NGS (Figures 2D and 2E). The hybrid *HBG1/2* promoters were specifically amplified using a nested PCR before performing the NGS. The characterization of the hybrid promoters enables to understand whether these novel promoters could also induce γ-globin expression. For pegRNA-195 the total frequency of precise and imprecise events was similar to that observed in the total *HBG1/2* promoter population, while pegRNA-115 showed a slightly lower overall editing frequency. Notably, the relative proportion of precise versus imprecise edits was increased in the hybrid promoters compared to the total promoters (Figures 2B–2E). In most cases, the incidence of indels was higher in hybrid *HBG1/2* promoters (compared to total promoters), except for pegRNA-195_S in RNA-transfected cells, which exhibited the highest frequencies of both precise and imprecise edits (Figures 2D and 2E). These results indicate that, despite the generation of *HBG1/2* 4.9-kb deletions using PEn based strategy, the resulting hybrid promoters could remain capable of activating γ-globin expression either by the installation of activating motifs or by the generation of indels that disrupt the repressor BSs (Figures 2D and 2E).

To favor the incorporation of precise edits and maximize γ-globin reactivation, we tested the treatment with DNA-PKc inhibitor (DNA-PKi, AZD7648) and Polθ inhibitor (Polθi, ART558) that inhibit NHEJ and alt-EJ, respectively (Figures S1C–S1F). The DNA-PKi was tested alone and in combination with Polθi in K562 cells (2i). The combined inhibition increased product purity and diminished the frequency of Indels, with the best-performing conditions being those using short pegRNA and RNA delivery (Figures S1C and S1D). Similar results were observed by NGS of the hybrid *HBG1/2* promoters (Figures S1E and S1F).

Finally, we tested our best-performing strategies (i.e., pegRNA-195_S and pegRNA-115_S) by RNA transfection in HSPCs obtained from patients with SCD. Editing efficiencies were low for the two strategies (with or without DNA-PKc and Polθ inhibitors) reaching only 6.4% and 2.7% of total prime edits for pegRNA-195_S and pegRNA-115_S, respectively (1.4% of precise edits for pegRNA-115_S; Figures 2F and 2G). In addition, the proportion of indels approached or exceeded the frequency of prime-editing events (Figures 2F and 2G). Prime-editing efficiency using a previously described engineered pegRNA (epegRNA) correcting the SCD mutation in the *HBB* gene averaged 60% (Figure S1G) in K562 (vs. 40% at the *HBG* promoters, Figures 2B and 2C) using RNA delivery. Notably, in 3 out of 5 donors, prime editing efficiency at *HBB* was consistent with the expected values,[37] confirming that HSPCs can be prime-edited using our protocol and reagents of comparable quality (Figures S1H). These results suggest that the *HBG1/2* promoters are particularly difficult to target with prime editing in HSPCs. However, the two strategies rely on different epegRNAs (different sequence and edits) and DNA-repair pathways, which can influence the editing outcome in HSPCs.

Cas9-HDR outperforms PEn in replacing the BCL11A BS by the TAL1:GATA1 motif, offering enhanced precision and reduced indels via DNA repair modulation

To increase the generation of precise edits, we developed a CRISPR-Cas9 HDR strategy (referred as Cas9-HDR) to replace the BCL11A repressor BS with the TAL1:GATA1 motif in the −115 region of the *HBG1/2* promoters. We used the sgRNA-115 that has been previously shown to disrupt the BCL11A BS using Cas9 nuclease and reactivate HbF.[20] We designed several single-stranded oligo-DNA nucleotides (ssODN) templates containing the partial BCL11A BS

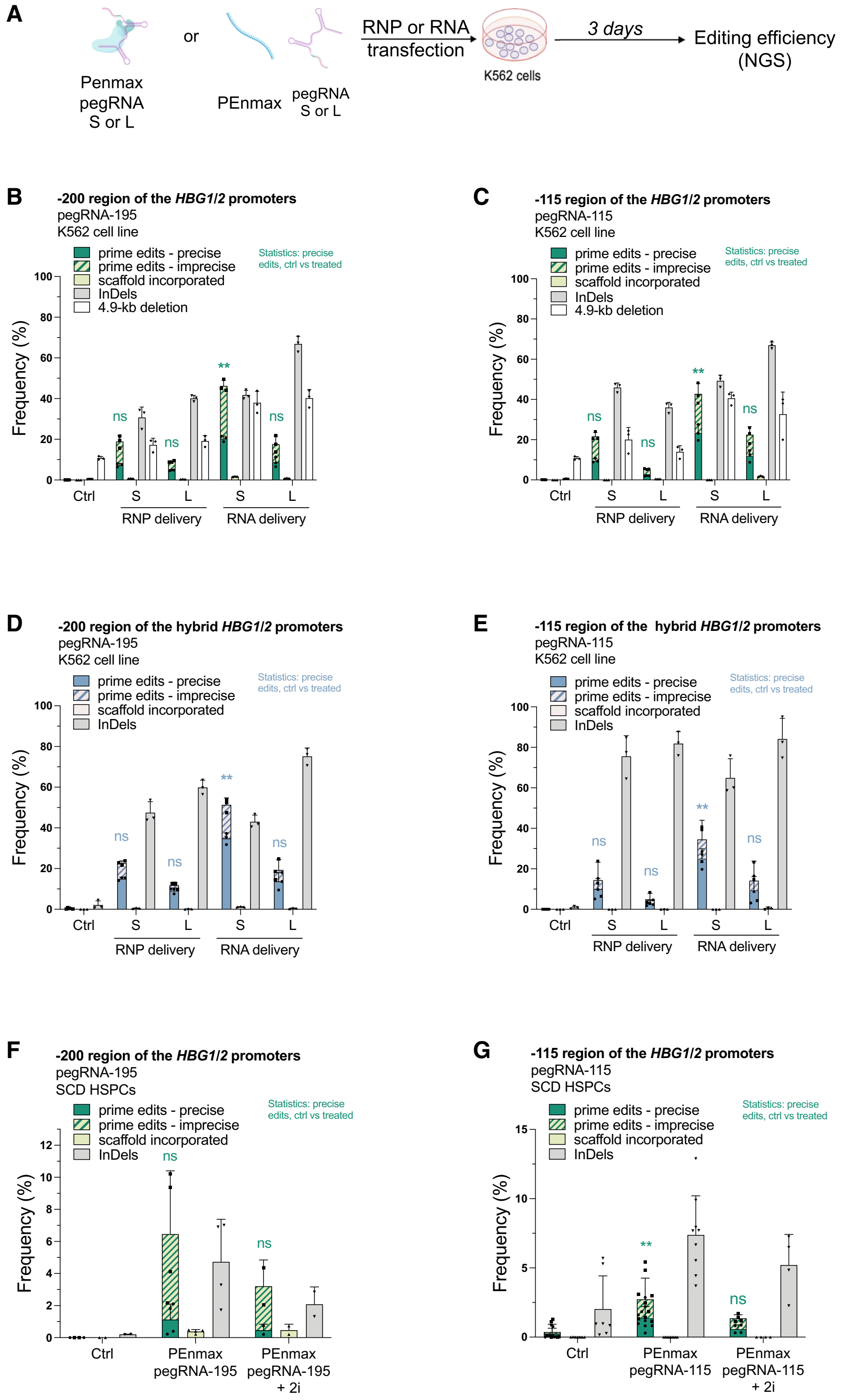

Figure 2. Optimization of the pegRNA constructs and the delivery method in K562 cells

(A) Experimental protocol used for PEnmax experiments in K562 cells. PEnmax and a pegRNA (with a short [S] or a long [L] homology tail) are delivered as RNA or ribonucleoprotein (RNP) in K562 cells. The prime editing efficiency is assessed by NGS 3 days after transfection and analyzed by CRISPResso2. Samples transfected with TE (Tris-EDTA) buffer were used as controls (Ctrl). (B and C) Percentage of the total *HBG1/2* promoters containing the total prime edits (precise, imprecise, scaffold incorporated) and indels induced by (B) pegRNA-195_S or pegRNA-115_L, or (C) pegRNA-115_S or pegRNA-115_L, delivered as RNA or RNP in K562 cells. We report also the frequency of the 4.9-kb deletion measured by ddPCR for control (Ctrl, TE-transfected cells) and prime-edited conditions. Bars represent the mean ± SD of 3 biological replicates. (D and E) Percentage of the hybrid *HBG1/2* promoters containing the prime edits (precise, imprecise, scaffold incorporated) and indels induced by (D) pegRNA-195_S or pegRNA-115_L or (E) pegRNA-115_S or pegRNA-115_L, delivered as RNA or RNP in K562 cells. Bars represent the mean ± SD of 3 biological replicates. (F and G) Percentage of total *HBG1/2* promoters containing the total prime edits (precise, imprecise, scaffold incorporated) and indels induced by (F) pegRNA-195_S or (G) pegRNA-115, delivered as RNA in HSPCs from patients with SCD treated or not with DNA repair inhibitors (2i). After electroporation, SCD HSPCs were cultured for 6 days in pre-activation medium before sequencing. The *HBG1/2* promoters were amplified and subjected to NGS. Data were analyzed with the CRISPResso2 webtool. Bars represent the mean ± SD of (F) 2–4 replicates of non-mobilized SCD CD34$^+$ cells from 2 different SCD donors and (G) 4 (2i treatment) to 9 (untreated) replicates of non-mobilized SCD CD34+ cells from 2 (2i treatment) to 6 (untreated) different SCD donors. (B–G) Statistical significance for "prime edits—precise" was assessed using a Kruskal-Wallis test followed by Dunn's multiple comparisons test, with the "Ctrl" condition used as the reference condition. $^{**}p < 0.01$. ns, not significant.

deletion, the TAL1:GATA1 sequence (Figure 1A). The HDR efficiency, when using ssODNs as DNA donor, is target dependent. Hence, we decided to design a wide range of homology arm length (i.e., ranging from 40 to 110 nt on each extremity of the ssODN) and symmetry to identify constructs that lead to efficient HDR when targeting the *HBG* promoters Figures 3A and 3B). As efficient HDR with ssODN was reported with homology arm lengths as short as 40 nt,[38] we designed symmetric ssODNs with homology arm lengths at 40, 50, and 60 nt (ssODN 1, 2, and 3). Additionally, we designed two ssODNs with asymmetric homology arms that were reported to increase HDR-mediated editing[39](60 and 110 nt for ssODN 4, and 110 and 60 nt for ssODN 5). ssODN were complementary to the (+) or (−) DNA strand with the target site being localized on the (+) strand (Figures 3A and 3B).

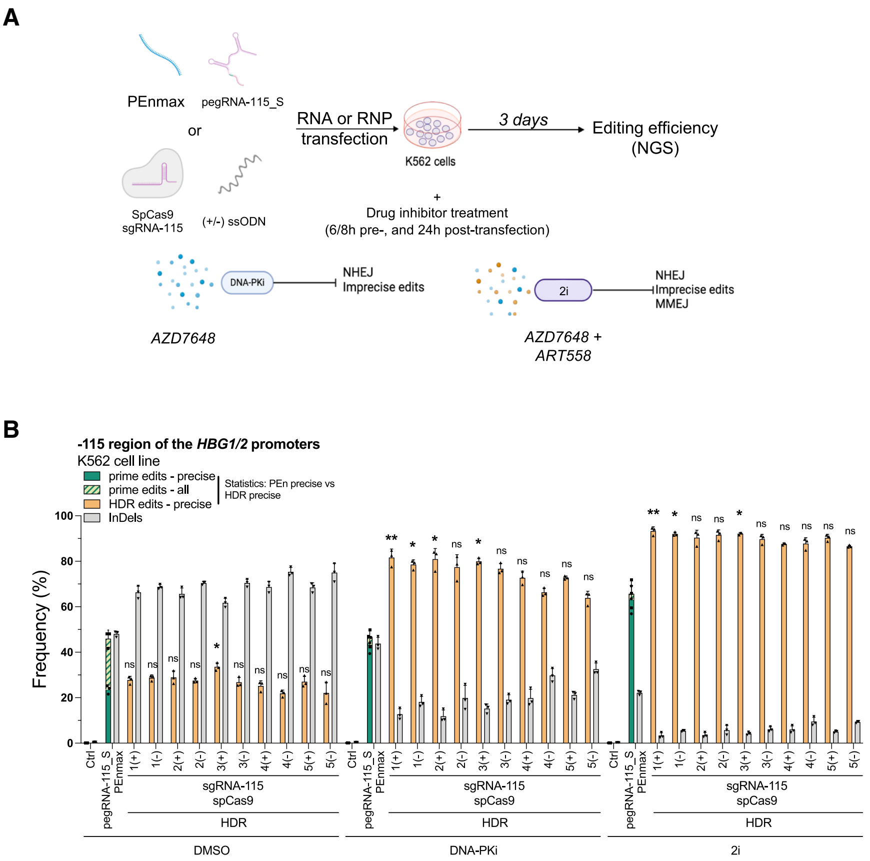

Figure 3. Design and screening of ssODNs to replace the BCL11A BS with the TAL1:GATA1 motif using a Cas9-HDR strategy in K562 cells

(A) Experimental protocol used for PEnmax and Cas9-HDR experiments in K562 cells. PEnmax and pegRNA-115_S were delivered as RNA and Cas9/sgRNA as RNP in K562 cells. Cells were treated with DMSO, 1 μM of AZD7648 (DNA-PKi), or with 1 μM of AZD7648 and 3 μM of ART558 (2i). The editing efficiency was assessed by NGS 3 days after transfection. Samples transfected with TE (Tris-EDTA) buffer were used as controls (Ctrl). (B) Percentage of total *HBG1/2* promoters containing the total prime edits (precise, imprecise), the HDR edits (precise), and indels induced by pegRNA-115_S and PEnmax, or sgRNA-115, Cas9, and ssODNs, in the presence or absence of different inhibitors. Bars represent the mean ± SD of 3 biological replicates. ssODNs were labeled numerically (1–5) to denote distinct designs, with (+) and (−) indicating the DNA strand targeted by the ssODN (Table S2). Statistical significance was assessed "prime edits—precise" using a Kruskal-Wallis test followed by Dunn's multiple comparisons test, with precise PEn editing used as the reference condition. *$p < 0.05$, **$p < 0.01$. ns, not significant.

First, we screened the different ssODNs in K562 cells by RNP transfection, a clinically approved method to deliver CRISPR-Cas9 reagents in HSPCs[17,40] (Figure 3A). In parallel, we compared the PEn/pegRNA115_S strategy using the best delivery method of the editing tools, i.e., RNA delivery (Figure 2D). Virtually all ssODNs generated the precise edits at a slightly higher level compared to the PEn strategy (Figure 3B). The Cas9-HDR strategies showed greater InDel frequencies in comparison to the PEn strategy (Figure 3B). To maximize γ-globin reactivation by reducing indels and favoring the incorporation of precise edits we tested DNA-PKi (AZD7648) and Polθi (ART558).[25,41] Polθi was not tested in the absence of DNA-PKi, as inhibition of Polθ alone does not to increase either HDR or homology-based PEn rates across multiple cell types.[25,42,43] The DNA-PKi led to greater increase of precise edits and reduction of indels in cells treated with Cas9-HDR compared to PEn-treated samples (Figure 3B). The combination of the two inhibitors (2i) further increased the product purity (i.e., the proportion of precise edits over indels) for both Cas9-HDR and PEn strategies (Figure 3B). The inhibition of the alt-EJ pathway is of particular importance in the −115 region that contains 8-bp MH motifs at the target site that may induce alt-EJ after DSB, thus generating at high frequency a 13-bp deletion, as we previously demonstrated[20] (Figure 1A). As an example, cells edited with Cas9, sgRNA-115 and ssODN (1+) showed ∼6%, ∼8%, and ∼1% of 13-bp deletion without inhibitors, with DNA-PKi alone, and with DNA-PKi in combination with Polθi, respectively. A similar trend was observed in PEn/pegRNA115_S-treated cells (∼9%, ∼31%, and ∼12%, without inhibitors, with DNA-PKi alone, and with DNA-PKi+Polθi, respectively). Among all ssODNs, we selected the ssODN (1+) that led to the highest editing efficiency (93.3%) and the lower InDel frequency (3.4%) after 2i treatment in K562 cells (Figure 3B).

The Cas9-HDR strategy efficiently reactivates γ-globin expression and corrects the sickling phenotype in RBCs differentiated from SCD HSPCs

We tested the Cas9-HDR strategy in primary cells derived from patients with SCD to evaluate if this combined approach (i.e., disruption of a repressor BS and insertion of two activator BSs) reactivates HbF expression to a higher level compared the sole disruption of the repressor BS.[20,22,23] HSPCs were transfected with RNPs containing sgRNA-115 and Cas9, with or without (1+) ssODN (Figure 4A). In addition, for the Cas9-HDR strategy, the cells were incubated with or without 2i for 24 h to promote precise edits and reduce indels, as shown in K562 cells (Figures 3B and 4A). Cell recovery, CD34 expression and viability of HSPCs were not affected upon editing; however, cell growth was reduced in edited samples, 6 days post-transfection (Figures S2A–S2C). In the absence of the ssODN, the DSB is mainly repaired by NHEJ and alt-EJ leading to the formation of indels (60.7%) and the disruption of the BCL11A BS, as previously demonstrated[20,22,23] (Figure 4B). In liquid erythroid cultures, we generated 12.3% and 17.7% of precise edits in Cas9-HDR-treated

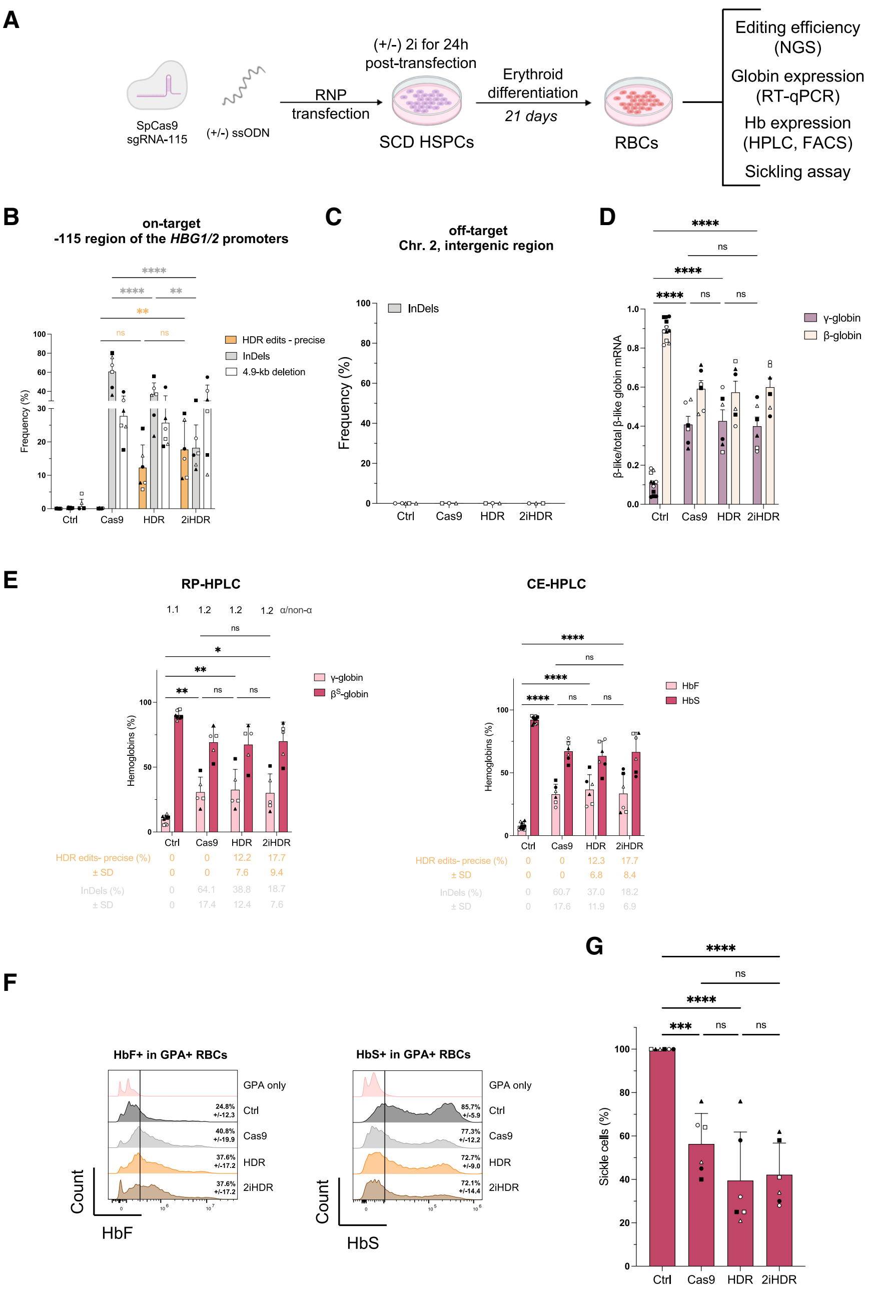

Figure 4. The replacement of the BCL11A BS by the TAL1:GATA1 motif reactivates HbF expression and corrects the sickling phenotype in RBCs differentiated from SCD HSPCs

(A) Experimental protocol used for the Cas9-based strategies in HSPCs derived from patients with SCD. Cas9 and sgRNA-115 were delivered as RNP complexes with or without ssODN (1+) in non-mobilized peripheral blood or bone marrow SCD HSPCs (n = 6 independent biological experiments; 5 different donors). After transfection, cells were treated for 24 h with 2i (2iHDR) or DMSO (as negative control), then differentiated for 21 days in mature RBCs and subjected to different assays. (B) Percentage of total *HBG1/2* promoters containing the HDR edits (precise) and indels, induced by sgRNA-115 and Cas9 +/− ssODN (1+) and 2i. The frequency of the 4.9-kb deletion was measured by ddPCR. Samples transfected with TE (Tris-EDTA) buffer or transfected with the Cas9 protein only were used as controls (Ctrl). Bars represent the mean ± SD of 6 independent biological experiments (5 different donors). Statistical significance was assessed for "prime edits—precise" and "indels" using two-way ANOVA followed by Tukey's multiple comparisons test. **p < 0.01, ****p < 0.0001. ns, not significant. (C) Editing at the main off-target site (chromosome 2, intergenic region), identified by GUIDE-seq[20] in control (Ctrl, TE- and transfected with the Cas9 protein), Cas9-, and HDR-treated conditions. Bars represent the mean ± SD of 3 independent biological experiments (2 different donors). (D) RT-qPCR analysis of β-like globin mRNA levels in patients' erythroblasts at day 13 of erythroid differentiation. β-like globin mRNA expression was normalized to α-globin mRNA. Bars represent the mean ± SD of 3 independent biological experiments (2 different donors). Statistical significance was assessed for "γ-globin" using two-way ANOVA followed by Tukey's multiple comparisons test. ****p < 0.0001. ns, not significant. (E) Expression of (left) $β^S$- and γ-globin chains, and (right) HbS and HbF measured by reverse-phased high performance liquid chromatography (RP-HPLC) and cation exchange high performance liquid chromatography (CE-HPLC), respectively, in RBCs differentiated from SCD HSPCs. We calculated the proportion of each β-like globin over the total β-like globin chains. Bars represent the mean ± SD of 3 independent biological experiments from 2 different donors. The ratio α/non-α globin is reported on top and HDR precise edits and indels are reported on the bottom. Statistical significance for "γ-globin" and "HbF" was assessed using two-way ANOVA followed by Tukey's multiple comparisons test. *p < 0.05, **p < 0.01, ****p < 0.0001. ns, not significant. (F) Flow cytometry histograms showing the percentage of (left) HbF- and (right) HbS-expressing cells in the GPA^+ population for samples stained only with the GPA antibody (GPA only) and for control (TE transfected) and edited samples. Frequency ±SD is indicated for 3 independent biological experiments from 2 different donors. (G) Frequency of sickled RBCs upon O_2 deprivation in control (Ctrl, TE transfected with the Cas9 protein) and edited samples. Cells were counted by a blinded observer for all conditions (>300 total randomly selected cells per condition). Bars represent the mean ± SD of 3 independent biological experiments (2 different donors). Statistical significance for "sickle cells" was assessed using two-way ANOVA followed by Tukey's multiple comparisons test. ***p < 0.001 ****p < 0.0001. ns, not significant. (B–G) n = 5 SCD donors, including 4 non-mobilized peripheral blood and 1 bone marrow sample (depicted as a black triangle), except for the left in Figure 2E (n = 3 SCD donors).

cells without and with 2i, respectively, exceeding the frequency obtained using the PEn strategy (Figures 2F and 4B). In addition, InDel frequency was significantly reduced upon 2i treatment (from 37.0% to 18.2%; Figure 4B). The frequency of *HBG1/2* 4.9-kb deletion was similar in all the samples (Figure 4B). Then, we assessed the off-target activity by sequencing the major off-target site of

sgRNA-115 (mapping to an intergenic region on chromosome 2) that we previously identified using genome-wide, unbiased identification of DSBs enabled by sequencing (GUIDE-seq).[20] Importantly, NGS did not reveal any indels in Cas9-, Cas9-HDR, or 2i-Cas9-HDR-treated conditions at the off-target site (Figure 4C).

Next, we differentiated control and treated cells in mature RBCs. The enucleation rate as well as the expression of erythroid markers was similar between control and edited samples, demonstrating no impact on the erythroid differentiation (Figures S3A–S3C). Quantitative reverse-transcription PCR (RT-qPCR), RP-HPLC, and CE-HPLC analyses showed a strong and significant γ-globin reactivation in all edited samples, at both RNA and protein levels (Figures 4D and 4E). Of note, the ratio between α and non-α globin chains was not altered in any of the samples (Figure 4E). Although the overall editing efficiency was lower in Cas9-HDR or 2i-Cas9-HDR-treated cells compared to Cas9 samples, we observed similarly high γ-globin and HbF levels across all three conditions (Figure 4E). Flow cytometry analysis also showed an increased frequency of HbF^+ cells and a decrease in the fraction of HbS-expressing cells in all edited samples (Figure 4F). These results suggest that precise edits generated by the Cas9-HDR approach are associated with great γ-globin reactivation (Figure 4E).

Finally, we incubated mature RBCs under hypoxic conditions to induce sickling (Figure 4G). All treatments led to significant decrease in the frequency of sickle cells, which was consistent with the similar γ-globin and HbF levels observed in bulk liquid erythroid cultures (Figure 4G).

A clonal analysis confirmed safety and efficacy of the HDR-based strategy replacing the BCL11A BS with the TAL1:GATA1-activating motif

HSPCs from patients with SCD were transfected with and without ssODN and 2i and plated in a semi-solid medium allowing erythroid (BFU-E) and granulocyte/monocyte (CFU-GM) differentiation at clonal level (colony-forming cell [CFC] assay; Figure 5A). Edited samples showed a similar clonogenicity potential, although 2i led to a reduction of erythroid colonies compared to control cells (Figure 5B). Editing efficiency in BFU-E and CFU-GM pools were similar to that obtained in liquid erythroid cultures; however, InDel frequency was reduced less effectively in BFU-E following 2i treatment (Figures 4B, 5C, and 5D).

Edited BFU-E bulk populations showed γ-globin reactivation (Figure 5E). To better characterize the proportion of precise edits and indels, we evaluated the genotype of single BFU-Es derived from Cas9-HDR- and 2i-Cas9-HDR-edited HSPCs (Figure 5F). We observed that 2i significantly reduced the proportion of BFU-Es carrying alleles containing indels (23.9%) compared to untreated cells (46.5%; Figure 5F). In addition, 2i favored the incorporation of the desired edits, leading to a higher proportion of clones harboring alleles with the precise edits in 1–4 *HBG1/2* promoters and clones with both precise edits and indels (Figure 5F).

To better evaluate the difference in γ-globin output associated with the combined HDR strategy and the sole Cas9-mediated disruption of the BCL11A BS, we measured γ-globin expression at mRNA level in single BFU-E clones harboring only the precise edits (obtained from both Cas9-HDR- and 2i-Cas9-HDR-edited HSPCs) or indels (Figures 5G and S4A). We observed a positive correlation between the frequency of the precise edits and γ-globin reactivation (R^2 = 0.68), while for InDel-carrying clones, γ-globin levels were more heterogeneous (R^2 = 0.18, Figure S4A). We then compared γ-globin expression in erythroid clones with a similar editing efficiency (≥80%, i.e., mainly clones with 4 edited promoters). Importantly, γ-globin reactivation was significantly higher in clones carrying the precise edits, i.e., the TAL1:GATA1 motif and the disruption of the BCL11A BS, than in clones harboring only indels disrupting the BCL11A repressor BS (Figure 5G).

Replacing the BCL11A BS with the TAL1:GATA1 motif produces more consistent HbF levels compared to introduction of the TAL1 BS alone

Finally, we compared the Cas9 and the Cas9-HDR approaches with a base editing strategy introducing the TAL1 BS at the position −175 (A>G) in the *HBG1/2* promoters[7,44] (Figure 6). We observed that γ-globin reactivation and HbF levels were not significantly different in bulk erythroid liquid cultures carrying the −175 TAL1 BS compared to Cas9 and Cas9-HDR treated samples (Figures 6A and 6B). It is noteworthy that desired edits in base-edited samples reached a mean of 64.2% (±11.4%), while Cas9-HDR and Cas9-2iHDR samples displayed only 14.8% (±8.3%) and 18.5% (±9.7%) of desired edits, respectively (Figure 6A). These results suggest that the combined strategy is more potent to reactivate gene expression compared to the introduction of the TAL1 activator BS alone. In fact, we observed a better correlation between the frequency of the precise edits and γ-globin reactivation in Cas9-HDR and Cas9-2iHDR conditions compared to erythroblasts carrying only indels or the TAL1 BS alone (Figure 6C).

To better evaluate the difference in γ-globin output associated with the Cas9, the Cas9-HDR and the base-editing strategies, we measured γ-globin mRNA expression in individual BFU-E harboring only indels, BFU-E carrying the TAL1:GATA1 motif (obtained from both Cas9-HDR- and 2i-Cas9-HDR-edited HSPCs), and BFU-E containing the TAL1 BS in position −175 (Figures 6D and 6E).

We observed a positive correlation between the frequency of the precise HDR edits and γ-globin reactivation (R^2 = 0.75), while for InDel- and TAL1 BS-carrying clones, γ-globin levels were more heterogeneous (R^2 = 0.073 and R^2 = 0.062, respectively, Figure 6D). We then compared γ-globin expression in erythroid clones with a similar editing efficiency (≥80%, i.e., mainly clones with 4 edited promoters). We confirmed that γ-globin reactivation was significantly higher in clones carrying the precise HDR edits, i.e., the TAL1:GATA1 motif and the disruption of the BCL11A BS, than in clones harboring only indels disrupting the BCL11A repressor BS (Figures 5G and 6E). In addition, BFU-E harboring the TAL1:GATA1 motif showed

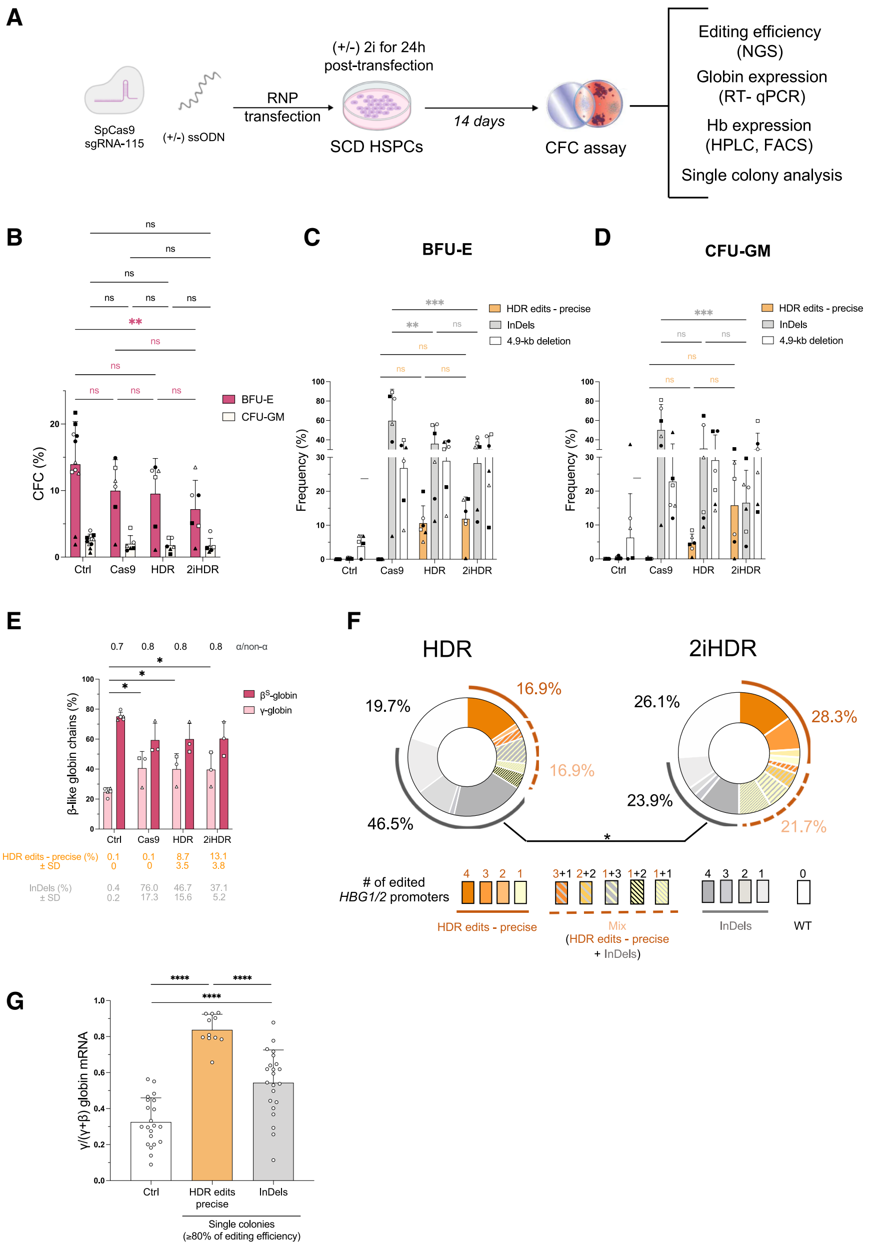

Figure 5. Viability, editing, and *HBG1/2* expression in progenitors derived from SCD HSPCs

(A) Experimental protocol used for the Cas9-based strategies in HSPCs derived from patients with SCD. Cas9 and sgRNA-115 were delivered as RNP complexes with or without ssODN (1+) in non-mobilized peripheral blood and bone marrow SCD HSPCs (n = 6 independent biological experiments; 5 different donors). After transfection, cells were treated for 24 h with 2i (2iHDR) or DMSO (as negative control), and then plated in a semi-solid medium allowing the growth of BFU-E and CFU-GM for 14 days. (B) BFU-E and CFU-GM frequencies for control (Ctrl, TE- transfected with the Cas9 protein only), Cas9-, and Cas9-HDR-treated conditions. Bars represent the mean ± SD of 6 independent biological experiments (5 different donors). Statistical significance for “BFU-E” and “CFU-GM” was assessed using two-way ANOVA followed by Tukey’s multiple comparisons test. $^{**}p < 0.01$. ns, not significant. (C and D) Percentage of total *HBG1/2* promoters in (C) BFU-E or (D) CFU-GM colonies, containing the HDR edits (precise), or indels in Ctrl (TE- and transfected with the Cas9 protein only), Cas9-, and Cas9-HDR-edited conditions ± the 2i, as evaluated by NGS. The frequency of the 4.9-kb deletions was measured by ddPCR. Bars represent the mean ± SD (n = 6 independent biological experiments; 5 different donors). Statistical significance for “HDR edits—precise” and “indels” was assessed using two-way ANOVA followed by Tukey’s multiple comparisons test. $^{**}p < 0.01$, $^{***}p < 0.001$. ns, not significant. (E) Expression of β- and γ-globin chains measured by RP-HPLC in BFU-E pools derived from SCD HSPCs. We calculated the proportion of each β-like globin over the total β-like globin chains. Bars represent the mean ± SD of 3 independent biological experiments (2 different donors). The ratio α/non-α globin is reported on top of the graph and HDR precise edits and indels are reported below the graph. Statistical significance for “γ-globin” was assessed using two-way ANOVA followed by Tukey’s multiple comparisons test. $^{*}p < 0.05$. (F) Frequency of BFU-E colonies carrying precise HDR edits, precise HDR edits and indels, indels, or WT promoters in HDR- or 2iHDR-treated conditions (n = 3 independent biological experiments (2 different donors); n = 71 and 46 single colonies, respectively). Edited colonies contain 1 to 4 γ-globin promoters either with only the HDR edits (precise, orange), only indels disrupting the BCL11A BS (gray), or a mix of all of HDR edits and indels (orange + gray). Statistical significance was assessed using two-way ANOVA with Sidak’s multiple comparison test. $^{*}p < 0.05$. (G) RT-qPCR analysis of γ-globin expression in single BFU-E derived from control (Ctrl, TE transfected with the Cas9 protein only) or edited cells. Edited colonies with ≥80% of editing efficiency were selected. The γ-globin mRNA expression was normalized to α-globin. Bars represent the mean ± SD of 11–22 single BFU-E from 3 independent biological experiments (2 different donors). Statistical significance was assessed using unpaired t test. $^{****}p < 0.0001$. (B–D) n = 5 SCD donors, including 4 non-mobilized peripheral blood and 1 bone marrow sample (black triangle).

more consistent γ-globin reactivation compared to colonies carrying only the TAL1 BS (Figure 6E).

DISCUSSION

Recent studies have demonstrated great potential in achieving high levels of therapeutic γ-globin expression to treat β-hemoglobinopathies. The exagamglogene autotemcel (known as Casgevy) is the first FDA-approved CRISPR-intervention that disrupts the *BCL11A* erythroid enhancer to treat patients with β-hemoglobinopathies.[17,40] However, the clinical study targeting the *BCL11A* enhancer showed variability in the extent of HbF reactivation among individuals, relatively low levels of Hb with HbS still

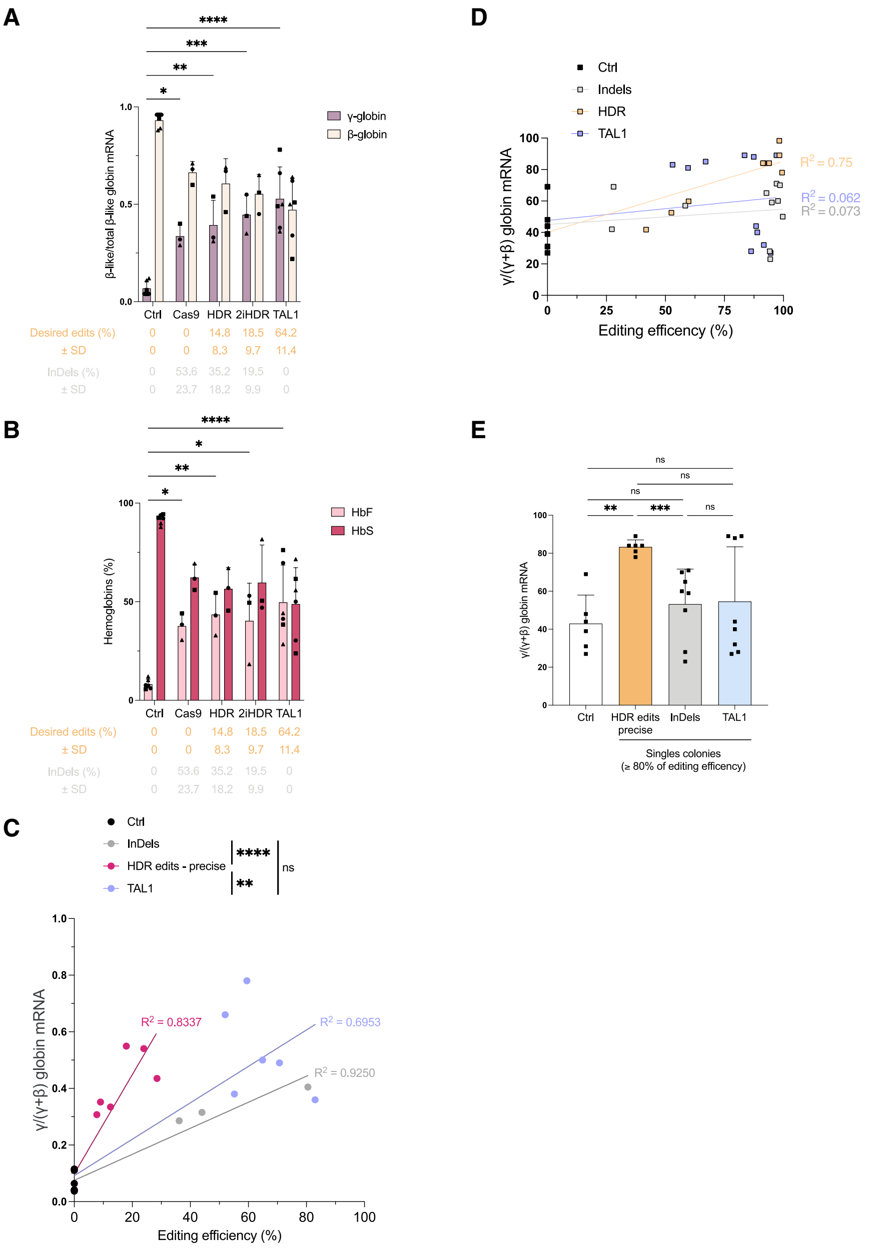

Figure 6. *HBG1/2* reactivation upon Cas9-HDR or base-editing treatment in erythroid cells derived from SCD HSPCs

(A) RT-qPCR analysis of β-like globin mRNA levels in patients' erythroblasts at day 13 of erythroid differentiation. β-like globin mRNA expression was normalized to α-globin mRNA. Bars represent the mean ± SD of 3 independent biological experiments (3 different donors). The frequency of desired edits (i.e., HDR precise edits for Cas9 strategies and A>G for base editing-mediated TAL1-binding site insertion using RNA or RNP delivery) and indels are reported on the bottom. Statistical significance for "γ-globin" was assessed using two-way ANOVA followed by Tukey's multiple comparisons test. *$p < 0.05$, **$p < 0.01$, ***$p < 0.001$, ****$p < 0.0001$. (B) Expression of HbS and HbF measured by CE-HPLC in RBCs differentiated from SCD HSPCs. We calculated the proportion of each β-like globin over the total β-like globin chains. Bars represent the mean ± SD of 3 independent biological experiments from 3 different donors. The frequency of desired edits (i.e., HDR precise edits for Cas9 strategies and A>G for base editing-mediated TAL1-binding site insertion) and indels are reported on the bottom. Statistical significance for "HbF" was assessed using two-way ANOVA followed by Tukey's multiple comparisons test. *$p < 0.05$, **$p < 0.01$, ****$p < 0.0001$. (C) Correlation between γ-globin mRNA expression and editing efficiency in patients' erythroblasts at day 13 of erythroid differentiation. γ-globin mRNA expression was normalized to α-globin mRNA. For HDR precise edits. (i.e., HDR and 2iHDR conditions), R^2 = 0.8337, Y = 0.01750*X + 0.09847; for Cas9 (i.e., indels), R^2 = 0.9250, Y = 0.004597*X + 0.07520; for A>G TAL1 binding site insertion, R^2 = 0.6953, Y = 0.0, 006432*X + 0, 09177 (simple linear regression). Statistical significance between 2 curves was assessed using linear regression: **$p < 0.01$, ****$p < 0.0001$. ns, not significant. (D) Correlation between γ-globin mRNA expression and editing efficiency in single erythroid BFU-E (n = 6 to 11 single BFU-E for each group). γ-globin mRNA expression was normalized to α-globin mRNA and expressed as a percentage of the total β- and γ-globin mRNA. For BFU-E containing the HDR precise edits, R^2 = 0.75, Y = 0.4512*X + 40.09; for BFU-E containing indels, R^2 = 0.073, Y = 0.09759*X + 45.05; for BFU-E containing A>G TAL1-binding site insertion, R^2 = 0.073, Y = 0.1497*X + 47.63 (simple linear regression). (E) RT-qPCR analysis of γ-globin expression in single BFU-E derived from control (Ctrl, TE-transfected colonies) or edited cells. Edited colonies with ≥80% of editing efficiency were selected. The γ-globin mRNA expression was normalized to α-globin. Bars represent the mean ± SD of 6–8 single BFU-E from 1 biological experiment (1 donor). Statistical significance was assessed using unpaired t test. **$p < 0.01$, ***$p < 0.001$. ns, not significant.

accounting for a large proportion of the total Hb and only modest correction of ineffective erythropoiesis,[17,40] the latter potentially due to the BCL11A role in erythroid development.[18,19] Recent work based on the disruption of the BCL11A repressor BS in the −115 region in the *HBG* promoters[22–24] showed significant increase in HbF levels (comparable to the Casgevy approach) with no detectable off-target activity.[15,45–49] This strategy is currently being tested in clinics in patients with β-hemoglobinopathies.[45–50] While the disruption of the BCL11A BS could recapitulate the effect of several benign HPFH mutations, in this study we envisioned a novel strategy to further boost HbF expression by simultaneously inserting a TAL1:GATA1 motif (known to recruit potent activators in erythroid cells) combined with the disruption of the BCL11A repressor BS. This could give a better selective advantage to erythroid cells, thus requiring a lower fraction of edited HSPCs to achieve a therapeutic benefit[51] (a scenario that is possible with an *in vivo* HSPC-targeting strategy[52]).

We first exploited the PEn system, which allows the incorporation of long DNA stretches in the genome[30] to replace the LRF or the BCL11A repressor BS, with a 18-bp long TAL1:GATA1 motif present in loci highly transcriptionally active during erythropoiesis.[31,32] The PEn_{max} yielded a higher proportion of total edits, including precise and imprecise edits, compared to the original PE2 system, reaching almost 40% of total edits at the −115 site of the *HBG1/2* promoters. Approximately half of these edits represented precise edits (i.e., precise deletion and insertion), while the remaining edits comprised partial insertion of the TAL1:GATA1 motif, or unintended incorporation of part of the pegRNA RTT/scaffold in the genome. Importantly, imprecise edits are also expected to cause γ-globin reactivation as they disrupt the repressor BS and/or facilitate the recruitment of either TAL1 or GATA1. We believe that PEn outperformed the classical PE system in terms of editing efficiency likely because of the kinetics of the resolution of the single-stranded breaks (SSBs) vs. DSBs.[53,54] Specifically, rapid cellular repair of SSB may prevent the incorporation of the reverse transcribed 3′ flap into the genome characteristic of PE strategy, whereas DSB repair leads to slower resolution, favoring the desired insert by the PEn system.

Due to the use of a nuclease, the PEn system led to the formation of indels that could nevertheless disrupt the repressor BS, mimicking the Cas9-mediated strategies developed for reactivating γ-globin.[20,22,23] The DSBs induced by the PEn system could also lead to the deletion of the 4.9-kb *HBG1-HBG2* intervening genomic region and the subsequent loss of the *HBG2* gene (due to the simultaneous cleavage of the two identical *HBG* promoters) at a high frequency that was, as expected, much higher compared to the original PE system. However, clonal analysis in Cas9-edited cells at the −115 region, showed that the hybrid *HBG* promoters reactivate HbF as long as the BCL11A repressor BS in the −115 region is disrupted at the junction site,[22,23] which was also observed in prime-edited samples.

The modulation of DNA repair pathways using DNA-PKi and/or Polθi increased the product purity and lowered indels, in line with our recent work. Interestingly, InDel frequency was reduced only by dual inhibition, likely because alt-EJ is upregulated upon NHEJ inhibition. Application of DNA-PKi alone or in combination with Polθi led to an increase of 4.9-kb deletion frequency, probably due to slower kinetics of DSB repair, which increases the possibility of simultaneous DSB formation and excision of intervening sequence.[55]

Notably, the type of reagents influenced the editing outcome. We observed significantly higher editing efficiency with mRNA-mediated PEn delivery in K562 cells, potentially due to sustained editor production.

This comprehensive optimization enabled the selection of optimal gene editing conditions to be tested in primary HSPCs from patients with SCD. Importantly, we demonstrated for the first time the successful replacement of the repressor BS (i.e., LRF or BCL11A) with an 18-nt long TAL1:GATA1 motif in the *HBG1/2* promoters of primary patient cells using PEn. Nevertheless, editing efficiency remained generally low and variable among the different donors, as we recently observed using PE and PEn.[34] Further optimization of the prime editing components and their delivery may be required to achieve clinically relevant editing frequencies.[56–58] Additionally, elucidating mechanisms underlying donor-to-donor variability may inform strategies to achieve more consistent editing outcomes.

We subsequently employed a Cas9-HDR approach to replace the BCL11A BS with the TAL1:GATA1 motif, achieving 90% of precise edits in K562 cells upon 2i treatment compared to 60% achieved with the PEn system. After optimizing protocol parameters in K562 cells, we compared the Cas9-HDR strategy with Cas9-mediated disruption of the BCL11A BS in HSPCs[20,22–24] obtaining up to 20% of targeted insertion events in primary SCD HSPCs with 2i. Importantly, replacing the BCL11A BS with the TAL1:GATA1 activating motif leads to higher and more consistent γ-globin levels than only disrupting the repressor BS (i.e., the therapeutic strategy currently in clinics) or creating only a TAL1 BS. These high HbF levels were sufficient to markedly alleviate the sickling phenotype.

No detectable editing was observed at the major predicted off-target site. Cas9-HDR edits did not interfere with cell viability and recovery, and erythroid differentiation, although reduction in progenitor numbers was noted. The DNA-PKi AZD7648 has been validated in clinical trials as standalone treatment or in combination with pegylated liposomal doxorubicin, showing potential safe profile in humans.[59] In the context of genome editing, it might compromise genomic stability (as recently proposed[60]) and it may require a more careful selection of gRNAs to minimize off-targets[61] and a comprehensive unbiased genotoxicity assessment to allow the clinical translation of this approach. Importantly, we recently demonstrated that co-inhibition of NHEJ and alt-EJ not only enhances editing efficiency and precision but also reduces off-target editing. Finally, this approach requires *in vivo* studies in immunodeficient mice to demonstrate engraftment, self-renewal, and multilineage differentiation of edited HSCs.

Overall, HDR-mediated gene editing was more efficient than prime editing. Prime editing has more design variables than Cas9-mediated HDR. It requires optimizing pegRNA spacer, PBS, RTT, and nicking sgRNA, whereas Cas9-mediated HDR is mainly influence by the choice of sgRNA and ssODN donor. This added design complexity is cell-type agnostic and applies to HSPCs. Mechanistically, PE outcomes depend on endogenous mismatch repair, which can suppress editing efficiency. Methodologically, we deliver PE components as RNA, while Cas9 is introduced as an RNP, and the length of pegRNAs—often exceeding the ∼150-nt limit of optimized RNA synthesis workflows—adds further limitations to the PE system. These factors collectively contribute to lower PE efficiency relative to Cas9-mediated HDR in HSPCs observed in our study.

In summary, this study highlights the limitation of prime editing tools for insertion of extended DNA sequences into the genome,

particularly in primary cells. Furthermore, our findings establish proof of concept that HDR-mediated insertion of TAL1:GATA1 motif combined with BCL11A BS disruption yields higher per cell HbF content than current Cas9-based approaches, suggesting a potential approach for the treatment of β-hemoglobinopathies. However, further optimization will be required to achieve higher frequencies and assay genetically modified cells *in vivo*.

MATERIALS AND METHODS

Cell line culture

Human erythroleukemia K562 cells were maintained at a concentration of 5×10^5 cells/ml in RPMI 1640 containing glutamine (Gibco) supplemented with 10% fetal bovine serum (Gibco), 2% HEPES (Life Technologies), 1% sodium pyruvate (Life Technologies), and 1% penicillin and streptomycin (Life Technologies) at 37°C and 5% CO_2.

$CD34^+$ cell purification and culture

We obtained human bone marrow and non-mobilized peripheral blood $CD34^+$ HSPCs from SCD patients. SCD samples eligible for research purposes were obtained from the Necker-Enfants malades Hospital (Paris, France). Written informed consent was obtained from all adult subjects. All experiments were performed in accordance with the Declaration of Helsinki. The study was approved by the regional investigational review board (reference: DC-2024-6899, CPP Ile-de-France II "Hôpital Necker-Enfants malades"). HSPCs were purified using the CD34 MicroBead Kit (Miltenyi Biotec). $CD34^+$ cells were thawed and cultured for 48 h at a concentration of 5×10^5 cells/ml in the "HSPC medium" containing StemSpan (STEMCELL Technologies) supplemented with penicillin/streptomycin (Gibco), 250 nM StemRegenin1 (STEMCELL Technologies), and the following recombinant human cytokines (PeproTech): human stem-cell factor (SCF) (300 ng/mL), FMS-like tyrosine kinase 3 ligand (Flt-3L) (300 ng/mL), thrombopoietin (TPO) (100 ng/mL), and interleukin-3 (IL-3) (60 ng/mL).

Plasmids

Plasmids used in this study include:

pMJ920 Cas9-GFP-expressing plasmid (Addgene #42234).

pCMV-PEmax (Addgene #174820).

pCMV-PEnmax (provided by AstraZeneca, Sweden) was generated by gene synthesis (Genscript). The PEnmax corresponds to PEmax with the H840A mutation reversed to the original histidine, restoring nuclease activity.

pU6-pegRNA-GG-acceptor (Addgene #132777).

The PEmax-SpRY and the PEnmax-SpRY plasmids were created by replacing the sequence encoding the PAM-interacting motif (PIM) of the Cas9 with the PIM-encoding sequence of the Cas9-SpRY.

- PIM sequence of the PEmax

5′GTGCAGACAGGCGGCTTCAGCAAAGAGTCTATCCTGCCC
AAGAGGAACAGCGATAAGCTGATCGCCAGAAAGAAGGAC
TGGGACCCTAAGAAGTACGGCGGCTTCGACAGCCCCACCG
TGGCCTATTCTGTGCTGGTGGTGGCCAAAGTGGAAAAGGG
CAAGTCCAAGAAACTGAAGAGTGTGAAAGAGCTGCTGGG
GATCACCATCATGGAAAGAAGCAGCTTCGAGAAGAATCCC
ATCGACTTTCTGGAAGCCAAGGGCTACAAAGAAGTGAAAA
AGGACCTGATCATCAAGCTGCCTAAGTACTCCCTGTTCGA
GCTGGAAAACGGCCGGAAGAGAATGCTGGCCTCTGCCGG
CGAACTGCAGAAGGGAAACGAACTGGCCCTGCCCTCCAAA
TATGTGAACTTCCTGTACCTGGCCAGCCACTATGAGAAGC
TGAAGGGCTCCCCCGAGGATAATGAGCAGAAACAGCTGTT
TGTGGAACAGCACAAGCACTACCTGGACGAGATCATCGAG
CAGATCAGCGAGTTCTCCAAGAGAGTGATCCTGGCCGACG
CTAATCTGGACAAAGTGCTGTCCGCCTACAACAAGCACCG
GGATAAGCCCATCAGAGAGCAGGCCGAGAATATCATCCAC
CTGTTTACCCTGACCAATCTGGGAGCCCCTGCCGCCTTCA
AGTACTTTGACACCACCATCGACCGGAAGAGGTACACCAG
CACCAAAGAGGTGCTGGACGCCACCCTGATCCACCAGAGC
ATCACCGGCCTGTACGAGACACGGATCGACCTGTCTCAGC
TGGGAGGTGAC3′

- PIM sequence of the PEmax-SpRY

5′GTGCAGACAGGCGGCTTCAGCAAAGAGTCTATCAGACCC
AAGAGGAACAGCGATAAGCTGATCGCCAGAAAGAAGGAC
TGGGACCCTAAGAAGTACGGCGGCTTCCTGTGGCCCACCG
TGGCCTATTCTGTGCTGGTGGTGGCCAAAGTGGAAAAGGG
CAAGTCCAAGAAACTGAAGAGTGTGAAAGAGCTGCTGGG
GATCACCATCATGGAAAGAAGCAGCTTCGAGAAGAATCCC
ATCGACTTTCTGGAAGCCAAGGGCTACAAAGAAGTGAAAA
AGGACCTGATCATCAAGCTGCCTAAGTACTCCCTGTTCGA
GCTGGAAAACGGCCGGAAGAGAATGCTGGCCTCTGCCAA
GCAGCTGCAGAAGGGAAACGAACTGGCCCTGCCCTCCAAA
TATGTGAACTTCCTGTACCTGGCCAGCCACTATGAGAAGC
TGAAGGGCTCCCCCGAGGATAATGAGCAGAAACAGCTGTT
TGTGGAACAGCACAAGCACTACCTGGACGAGATCATCGAG
CAGATCAGCGAGTTCTCCAAGAGAGTGATCCTGGCCGACG
CTAATCTGGACAAAGTGCTGTCCGCCTACAACAAGCACCG
GGATAAGCCCATCAGAGAGCAGGCCGAGAATATCATCCAC
CTGTTTACCCTGACCAGACTGGGAGCCCCTAGAGCCTTCA
AGTACTTTGACACCACCATCGACCCCAAGCAGTACAGAAG
CACCAAAGAGGTGCTGGACGCCACCCTGATCCACCAGAGC
ATCACCGGCCTGTACGAGACACGGATCGACCTGTCTCAGC
TGGGAGGTGAC3′

All plasmids were optimized to contain a T7 promoter compatible with the TriLink's CleanCap-AG Cap1 analog and a 3′UTR that we previously described.[36]

All pegRNAs were generated by gene synthesis and cloned in pMA vector together with an upstream U6 promoter (GeneArt).

PEnmax and ABE8e-SpCas9-NGG mRNA *in vitro* transcription

Capped PEnmax mRNA was generated following T7 directed *in vitro* transcription using a linearized PEnmax DNA template. The *in vitro* transcription reaction produced mRNA fully modified, replacing uridines with N1-Methyl-pseudouridine, and cap-1 capped using TriLink's CleanCap-AG Cap1 analog. The mRNA was subsequently column purified using MEGAClear transcription clean-up kit (Thermo Fisher Scientific) and mRNA purity was analyzed using a fragment analyzer (Agilent).

Synthetic pegRNAs, sgRNAs, and ssODN constructs

The PBS and RTT were manually designed for all pegRNAs, for which we selected the recommended PBS length of 13-nt, except for pegRNA-195 that was designed using the Prime Design webtool with a 16-nt-long PBS. All pegRNAs carry an RTT (with a length ranging from 48- to 55-nt) containing a total or partial deletion of the LRF or the BCL11A BSs, the 18-bp long TAL1:GATA1 sequence, and a 30-nt long homology tail (or 36-nt long for the pegRNA-195 as defined with the Prime Design webtool). HDR ssODNs were manually designed to target (+) or (−) DNA strand. epegRNA and ngRNA correcting the SCD mutation were selected from Everette et al., 2023.[37]

Chemically modified synthetic pegRNAs, sgRNAs and ssODN were ordered from Integrated DNA Technologies (IDT). Each construct harbored 2′-*O*-methyl analogs and 3′-phosphorothioate non hydrolyzable linkages at the first three 5′ and 3′ nucleotides. All pegRNA and and ssODNs sequences used in this study are respectively listed in Tables S1 and S2. The target sequence of the sgRNA-115 is 5′-CTTGTCAAGGCTATTGGTCA**AAG**-3′, with the protospacer adjacent motif highlighted in bold.[20]

Protein expression and purification

Cas9 was purchased from IDT (Alt-R S.p. Cas9 Nuclease V3). The sequence of PEn with a C-terminal his tag was cloned into pET24a. The expression plasmid was then transformed into *Escherichia coli* BL21lDE3 Star (Thermo Fisher Scientific) for use in protein production. Autoinduction protocol[62] was used for the over-production of PEn. Essentially, the culture was first grown overnight at 37°C, before inoculation with 800 mL of ZYP autoinduction media, which was then grown at 37°C with shaking until OD600 reached about 1–2. The temperature was then lowered to 18°C and the culture was grown for a further 24 h, before harvesting the cells by centrifugation. Cell pellets were stored at −80°C until further use. The cell pellets were then resuspended in 20 mM HEPES, pH 7.5, 500 mM NaCl, 1 mM DTT, 10% glycerol, and lysed by one pass through an Emulsiflex C3 (Avestin). The lysate was clarified by centrifugation at 20,000 g for 20 min. The supernatant was supplemented with 10 mM imidazole and the lysate was loaded onto a 5 mL HiTrap column (Cytiva) equilibrated in the same buffer. The column was washed with 20 column volumes of 20 mM HEPES pH, 7.5, 500 mM NaCl, 1 mM DTT, 10% glycerol, 20 mM imidazole, before elution with 300 mM imidazole. The eluted protein was diluted to about 200 mM NaCl, before further purification on a 5 mL HiTrap Heparin SP column (Cytiva). Finally, the protein was further purified by size exclusion chromatography on a Superdex 200 (26/60) column (Cytiva) equilibrated in a buffer consisting of 20 mM HEPES, pH 7.5, 300 mM NaCl, 1 mM DTT, and 10% glycerol. The peak containing the PEn protein was pooled, concentrated to 10 mg/mL, flash-frozen in liquid nitrogen and stored at −80°C until required.

The ABE8e-SpCas9-NGG proteins were expressed in *E. coli* BL21 Star (DE3) cells using the pD881-SR plasmid. Cells were grown in Terrific Broth at 37°C to an OD600 of ∼1.5, cold-shocked, and induced with 0.8% l-rhamnose at 18°C for 24 h. Harvested cells were lysed via homogenization, and the clarified lysate was subjected to heparin affinity chromatography using a tris pH 7.5 based buffer system using 480 mM salt and 10% glycerol. The base editor protein was eluted using a linear gradient of 20CV salt to a final concentration of 650 mM. The eluted protein was further purified by cation exchange chromatography (HiTrap SP HP) using an AKTA FPLC system with a salt gradient. Fractions containing the desired protein were pooled, concentrated using 100-kDa cutoff filters, and quantified via BCA assay to a final stock concentration. Detailed buffer compositions and full purification procedures except for the affinity step are described previously.[7]

Plasmid transfection

K562 cells (10^6 cells/condition) were transfected with 3.6 μg of PEmax or PEnmax and 1.2 μg of the pegRNA-containing plasmid using AMAXA Cell Line Nucleofector Kit V (VCA- 1003, Lonza) and U-16 program (Nucleofector 2b, Lonza). Cells transfected with TE buffer were used as negative controls.

RNA transfection

K562 cells (2×10^5 cells/condition) were electroporated with 2 μg of PEn mRNA and 100 pmol of synthetic pegRNA using the SF Cell Line 96-well Nucleofector Kit (Lonza) and the FF-120 pulse code (Nucleofector 4D; Lonza). Post-electroporation, cells were incubated at room temperature for 10 min without any disturbance and were thence transferred into pre-warmed medium in 96-well plates and incubated at 37°C with 5% CO_2 for 72 h before cell collection and DNA extraction. Cells transfected with TE buffer were used as negative controls.

RNP and ssODN transfection

RNP complexes were assembled at room temperature using a final concentration of Cas9 at 3 μM, PEn at 2.7 μM, and synthetic sgRNAs/pegRNAs at 5 μM. K562 cells (2×10^5 cells/condition) were transfected with RNP complexes and ssODNs at 10 μM in the final transfection reaction, using the SF Cell Line 96-well Nucleofector Kit (Lonza), and the FF-120 pulse code (Nucleofector 4D) in the presence of a transfection enhancer (IDT). $CD34^+$ HSPCs (2×10^5 cells/condition) were transfected with RNP complexes and ssODNs at 10 μM in the final transfection reaction, using the P3 Primary Cell 4D-Nucleofector X Kit S (Lonza) and the CA-137 program (Nucleofector 4D) in the presence of a transfection

enhancer (IDT). Post-electroporation, cells were incubated at room temperature for 10 min without any disturbance and were thence transferred into pre-warmed medium in 96-well plates and incubated at 37°C with 5% CO_2 for 72 h for K562 cells or 6 days for HSPCs before cell collection and DNA extraction. Non-transfected cells or cells transfected with TE buffer or with enzyme only were used as negative controls.

Small molecule compounds and drug treatment

DNA-PKc inhibitor AZD7648 was provided by AstraZeneca (Gothenburg, SE). PolΘ inhibitor, ART558 was purchased from MedChemExpress (HY-141520). All compounds were dissolved in dimethyl sulfoxide (DMSO) at a stock concentration of 10 mM. K562 cells were cultured for 24 h post-transfection in a medium containing 1 μM of AZD7648, or in combination with 3 μM ART558 (2i) when indicated. $CD34^+$ cells were cultured in a medium containing 1 μM of AZD7648 and 3 μM ART558 (2i), for 24 h post-transfection. All inhibitor treatments were followed by a cell wash and culture in control medium.

HSPC counting

At day 0, immediately following nucleofection, 1/6 of the total cell volume was resuspended at a concentration of 1×10^6 cells/mL and seeded into pre-activation medium. Cell counts were performed on days 1, 4, and 6 using Countess Cell Counting Chamber Slides and the Countess 3 Automated Cell Counter (Thermo Fisher Scientific).

HSPC differentiation

Transfected $CD34^+$ HSPCs were differentiated into mature RBCs using a three-phase erythroid differentiation protocol, as previously described.[34,63] During the first phase (day 0 to day 6), cells were cultured in a basal erythroid medium supplemented with 100 ng/mL recombinant human SCF (PeproTech), 5 ng/mL recombinant human IL3 (PeproTech), 3 IU/mL EPO Eprex (Janssen-Cilag), and 10^{-6}M hydrocortisone (Sigma). During the second phase (day 6 to day 9), cells were co-cultured with MS5 stromal cells in the basal erythroid medium supplemented with 3 IU/mL EPO Eprex (Janssen-Cilag). During the third phase (day 9 to day 20), cells were co-cultured with stromal MS5 cells in a basal erythroid medium without cytokines. Heat-inactivated human AB serum was added during the third phase of the differentiation (10%; day 13 to day 20). Erythroid differentiation was monitored by flow cytometry analysis of CD36, CD71, GPA, Band3, and α4-Integrin erythroid surface markers and of enucleated cells using the DRAQ5 double-stranded DNA dye. 7AAD was used to identify live cells.

CFC assay

$CD34^+$ HSPCs were plated at a concentration of 500 cells/mL in a methylcellulose-based medium (GFH4435, STEMCELL Technologies) under conditions supporting erythroid and granulocyte/monocyte differentiation. BFU-E and CFU-GM colonies were counted after 14 days. Colonies were randomly picked and collected as bulk populations (n = 25 colonies) to evaluate the editing efficiency, globin expression by RT-qPCR and RP-HPLC. BFU-Es were randomly picked and collected as single colonies (around 24 colonies per sample) to evaluate the efficiency and globin expression by RT-qPCR.

Genomic DNA extraction and sequencing for on- and off-target analysis

Genomic DNA from K562 cells was extracted using Quick Extract solution (LGC Biosearch Technologies) or the PureLink Genomic DNA Mini Kit (Invitrogen), 3 days post-transfection, following manufacturer's instructions. Genomic DNA from $CD34^+$ was harvested 6 days post-transfection using the PureLink Genomic DNA Mini Kit (Invitrogen) following the manufacturer's instructions.

To evaluate the editing efficiency in K562 cells' screening experiments (Figure S1A), *HBG1/2* promoters' on-target sites were PCR amplified, with 5′-AAAAACGGCTGACAAAAGAAGTCCTGGTAT-3′ forward primer and 5′-ATAACCTCAGACGTTCCAGAAGCGAGTGTG-3′ reverse primer, using the Recombinant Taq DNA Polymerase (Thermo Fisher Scientific) according to the manufacturer's instructions, and subjected to Sanger sequencing. The precise prime edits, i.e., expected insertion size, and the indels were evaluated using the TIDE software (http://shinyapps.datacurators.nl/tide/).

The *HBG* promoters were amplified with 5′-GGAATGACTGAATCGGAACAAGG-3′ forward primer and 5′-CTGGCCTCACTGGATACTCT-3′ reverse primer for pegRNA-115 and sgRNA-115 targeting samples. The hybrid *HBG1/2* promoter was amplified by a nested PCR with 5′-GTTTTAAAACAACAAAAATGAGGGAAAGA-3′ forward primer and 5′-GTTGCTTTATAGGATTTTTCACTACAC-3′ reverse primer using Phusion Flash High-Fidelity 2x Mastermix (F548, Thermo Scientific) in a 30 μL reaction, containing 1.0 μL of genomic DNA extract and 0.2 μM of target-specific primers (98°C for 3 min, followed by 40 cycles of 98°C for 10 s, 60°C for 5 s, and 72°C for 18 s), followed by amplification using pegRNA-115 and sgRNA-115 targeting sample primers. Amplicons were generated with Phusion Flash High-Fidelity 2x Mastermix (F548, Thermo Scientific) in a 15 μL reaction, containing 1.5 μL of genomic DNA extract or 1st PCR product (for nested PCR) and 0.2 μM of target-specific primers with barcodes and NGS adapters. PCR cycling conditions for Phusion Flash High-Fidelity 2x Mastermix were: 98°C for 3 min, followed by 30 cycles of 98°C for 10 s, 60°C for 20 s, and 72°C for 30 s and final elongation at 72°C for 2 min. All amplicons were purified using HighPrep PCR Clean-up System (MagBio Genomics). Size, purity, and concentration of amplicons were determined using a fragment analyzer (Agilent). To add Illumina indexes to the amplicons, samples were subjected to a second round of PCR. Indexing PCR was performed using KAPA HiFi HotStart Ready Mix (Roche), 0.067 ng of PCR template and 0.5 μM of indexed primers in the total reaction volume of 25 μL. PCR cycling conditions were 72°C for 3 min, 98°C for 30 s, followed by 10 cycles of 98°C for 10 s, 63°C for 30 s, and 72°C for 3 min, with a final extension at 72°C for 5 min. Samples were purified with the HighPrep PCR Clean-up System (MagBio Genomics) and analyzed using a fragment analyzer (Agilent).

Samples were quantified using a Qubit 4 Fluorometer (Life Technologies) and subjected to sequencing using Illumina NextSeq system according to manufacturer's instructions. Table S3 lists the primers used for on- and off-target analysis by deep sequencing.

Bioinformatic analysis

Demultiplexing of the NGS sequencing data was performed using bcl2fastq software. The fastq files were analyzed using CRISPResso2 v.2.2.12 in the HDR or prime editing mode. Detailed parameters are listed in Table S4.

RT-qPCR

Total RNA was extracted from HSPCs, BFU-E pools and erythroblasts (day 13) using the RNeasy micro kit (QIAGEN), and single colonies using the AllPrep DNA/RNA kit (QIAGEN). RNA was treated with DNase using the DNase I kit (Invitrogen), following the manufacturer's instructions. Mature transcripts were reverse transcribed using SuperScript III First Strand Synthesis System for RT-qPCR (Invitrogen) with oligo (dT) primers. RT-qPCR was performed using the iTaq universal SYBR Green master mix (Bio-Rad) and the CFX384 Touch Real-Time PCR Detection System (Bio-Rad). Table S5 lists the primers used for RT-qPCR analysis.

Evaluation of the 4.9-kb deletion

ddPCR was performed using a primer/probe mix (Bio-Rad) to quantify the frequency of the 4.9-kb deletion. Control primers annealing to *HBG2* were used as DNA loading control. ddPCR was performed using the ddPCR supermix (no dUTP) to quantify the frequency of *HBG2* loss over *HBG1* in each treated sample. Data were acquired through QX200 analyzer (Bio-Rad) and results were analyzed with QuantasoftTM Analysis Pro (Bio-Rad). Table S6 lists the primers used for the ddPCR analysis.

Flow cytometry analysis

HSPCs were stained with a PE-conjugated Annexin V antibody (1/10, 640947, BioLegend), an APC-Cy7-conjugated anti-CD34 antibody (1/25, 343514, BioLegend), and Live/Dead Aqua fixable viability dye (L34957, Invitrogen). Cells (20,000–50,000 per condition) were washed in Annexin V Binding Buffer (422201, BioLegend) prior to staining. We performed Annexin V and Live/Dead Aqua staining to define four populations: live (Annexin V^-/$Aqua^-$), early apoptotic (Annexin V^+/$Aqua^-$), late apoptotic (Annexin V^+/$Aqua^+$), and necrotic (Annexin V^-/$Aqua^+$) cells. Flow cytometry analyses were performed on an Agilent Penteon cytometer (Agilent Technologies). Data were analyzed using FlowJo (BD Biosciences) software.

HSPC-derived erythroid cells were stained with an antibody recognizing GPA erythroid surface marker (1/100 PE-Cy7 conjugated anti-GPA antibody, 563666, BD Biosciences), then fixed with 0.05% cold glutaraldehyde and permeabilized with 0.1% Triton X-100. After fixation and permeabilization, cells were stained with either an antibody recognizing HbF (1/5 FITC-conjugated anti-HbF antibody, clone 2D12 552829 BD Biosciences), or an antibody recognizing HbS (1/20 anti-HbS antibody, H04181601, BioMedomics) followed by the staining with a secondary antibody recognizing rabbit IgG (1/200 BV421-conjugated anti-rabbit IgG, 565014, BD Biosciences). Flow cytometry analysis of CD36, CD71, GPA, BAND3, and α4-Integrin erythroid surface markers was performed using a V450-conjugated anti-CD36 antibody (1/100 561535, BD Horizon), a FITC-conjugated anti-CD71 antibody (1/100 555536, BD Biosciences), a PE-Cy7 conjugated anti-GPA antibody (1/100 563666, BD Biosciences), a PE-conjugated anti-BAND3 antibody (1/50 9439, IBGRL) and an APC-conjugated anti-CD49d antibody (1/20 559881, BD Biosciences). Flow cytometry analysis of enucleated or viable cells was performed using double-stranded DNA dyes (DRAQ5, 65-0880-96, Invitrogen and 7AAD, 559925, BD, respectively). Flow cytometry analyses were performed using Novocyte flow cytometers (Agilent Technologies). Data were analyzed using the FlowJo (BD Biosciences) software.

Statistics

Data visualization and statistical analysis were conducted using GraphPad Prism 9 (GraphPad Software, Inc.). Figure legends contain information on statistical tests, sample sizes, and *p* values. Briefly, we used Shapiro-Wilk test to evaluate if data follow a Gaussian distribution. For comparison between two groups, we used the parametric unpaired *t* test if data followed a Gaussian distribution and the non-parametric Mann-Whitney test if data did not follow a Gaussian distribution. For comparison between more than two groups, we used Kruskal-Wallis test followed by Dunn's multiple comparisons, or a two-way ANOVA followed by Tukey's multiple comparisons test. No data were excluded from the analyses. No statistical method was used to predetermine sample size. The experiments were not randomized. The investigators were not blinded during experiments and outcome assessment.

DATA AND CODE AVAILABILITY

All data supporting the findings of this study are available within the paper and its supplemental information.

ACKNOWLEDGMENTS

We thank Dr. Sandra Manceau for the collection of the blood samples and the technological core facilities of the SFR Necker (Inserm US24/CNRS UAR3633) including the flow cytometry platform and the imaging facility. This work was supported by state funding from the French National Research Agency (Agence Nationale de la Recherche; ANR-10-IAHU-01 and ANR-22-CE17-0028 PEMGeT), by Region Île-de-France in the framework of DIM BioConvS, the European Commission (HORIZON-PathFinder EdiGenT grant no. 101070903), the COST European Cooperation in Science and Technology (the COST Action Gene Editing for the treatment of Human Diseases, CA21113) and ERDERA, which has received funding from the European Union's Horizon Europe research and innovation program under grant agreement N°101156595. This study is part of the Université Paris Cité IdEx #ANR-18-IDEX-0001\ funded by the French Government through its "Investments for the Future" program.

AUTHOR CONTRIBUTIONS

A.C., conceptualization, formal analysis, investigation, and writing – original draft; P.A., conceptualization, formal analysis, investigation, and writing – original draft; M.B.D., P.M., E.R., and P.G., formal analysis and investigation; M.F., data curation formal analysis, investigation; M.P., formal analysis, and investigation; M.R., A.S.C., and J.S.Y., resources; M.M., conceptualization, funding acquisition, supervision, validation, and Verification, writing – review and editing; M.B., conceptualization, project administration,

supervision, validation, verification, writing – original draft, and writing – review and editing; A.M., conceptualization, funding acquisition, project administration, supervision, validation, verification, and writing – review and editing.

DECLARATION OF INTERESTS

P.A., P.G., M.F., M.P., and M.M. are employees of AstraZeneca and may be AstraZeneca shareholders.

SUPPLEMENTAL INFORMATION

Supplemental information can be found online at https://doi.org/10.1016/j.omtn.2026.102974.

REFERENCES

1. Thein, S.L. (2018). Molecular basis of β thalassemia and potential therapeutic targets. Blood Cells. Mol. Dis. *70*, 54–65. https://doi.org/10.1016/j.bcmd.2017.06.001.
2. Piel, F.B., Steinberg, M.H., and Rees, D.C. (2017). Sickle Cell Disease. N. Engl. J. Med. *376*, 1561–1573. https://doi.org/10.1056/NEJMra1510865.
3. Taher, A.T., Weatherall, D.J., and Cappellini, M.D. (2018). Thalassaemia. Lancet Lond. Engl. *391*, 155–167. https://doi.org/10.1016/S0140-6736(17)31822-6.
4. Locatelli, F., Lucarelli, B., and Merli, P. (2014). Current and future approaches to treat graft failure after allogeneic hematopoietic stem cell transplantation. Expert Opin. Pharmacother. *15*, 23–36. https://doi.org/10.1517/14656566.2014.852537.
5. Forget, B.G. (1998). Molecular Basis of Hereditary Persistence of Fetal Hemoglobin. Ann. N. Y. Acad. Sci. *850*, 38–44. https://doi.org/10.1111/j.1749-6632.1998.tb10460.x.
6. Wienert, B., Martyn, G.E., Kurita, R., Nakamura, Y., Quinlan, K.G.R., and Crossley, M. (2017). KLF1 drives the expression of fetal hemoglobin in British HPFH. Blood *130*, 803–807. https://doi.org/10.1182/blood-2017-02-767400.
7. Mayuranathan, T., Newby, G.A., Feng, R., Yao, Y., Mayberry, K.D., Lazzarotto, C.R., Li, Y., Levine, R.M., Nimmagadda, N., Dempsey, E., et al. (2023). Potent and uniform fetal hemoglobin induction via base editing. Nat. Genet. *55*, 1210–1220. https://doi.org/10.1038/s41588-023-01434-7.
8. Doerfler, P.A., Feng, R., Li, Y., Palmer, L.E., Porter, S.N., Bell, H.W., Crossley, M., Pruett-Miller, S.M., Cheng, Y., and Weiss, M.J. (2021). Activation of γ-globin gene expression by GATA1 and NF-Y in hereditary persistence of fetal hemoglobin. Nat. Genet. *53*, 1177–1186. https://doi.org/10.1038/s41588-021-00904-0.
9. Martyn, G.E., Wienert, B., Yang, L., Shah, M., Norton, L.J., Burdach, J., Kurita, R., Nakamura, Y., Pearson, R.C.M., Funnell, A.P.W., et al. (2018). Natural regulatory mutations elevate the fetal globin gene via disruption of BCL11A or ZBTB7A binding. Nat. Genet. *50*, 498–503. https://doi.org/10.1038/s41588-018-0085-0.
10. Coleman, M.B., Adams, J.G., Steinberg, M.H., Plonczynski, M.W., Harrell, A.H., Castro, O., and Winter, W.P. (1993). GγAγ(β+) hereditary persistence of fetal hemoglobin: The Gγ − 158 C → T mutation in cis to the − 175 T → C mutation of the Aγ-globin gene results in increased Gγ-globin synthesis. Am. J. Hematol. *42*, 186–190. https://doi.org/10.1002/ajh.2830420209.
11. Lieber, M.R. (2010). The Mechanism of Double-Strand DNA Break Repair by the Nonhomologous DNA End-Joining Pathway. Annu. Rev. Biochem. *79*, 181–211. https://doi.org/10.1146/annurev.biochem.052308.093131.
12. Seol, J.-H., Shim, E.Y., and Lee, S.E. (2018). Microhomology-mediated end joining: Good, bad and ugly. Mutat. Res. Mol. Mech. Mutagen. *809*, 81–87. https://doi.org/10.1016/j.mrfmmm.2017.07.002.
13. Chang, H.H.Y., Pannunzio, N.R., Adachi, N., and Lieber, M.R. (2017). Non-homologous DNA end joining and alternative pathways to double-strand break repair. Nat. Rev. Mol. Cell Biol. *18*, 495–506. https://doi.org/10.1038/nrm.2017.48.
14. Kanter, J., Thompson, A.A., Pierciey, F.J., Hsieh, M., Uchida, N., Leboulch, P., Schmidt, M., Bonner, M., Guo, R., Miller, A., et al. (2023). Lovo-cel gene therapy for sickle cell disease: Treatment process evolution and outcomes in the initial groups of the HGB-206 study. Am. J. Hematol. *98*, 11–22. https://doi.org/10.1002/ajh.26741.
15. Zeng, J., Nguyen, M.A., Liu, P., da Silva, L.F., Levesque, S., Lin, L.Y., Justus, D.G., Petri, K., Clement, K., Porter, S.N., et al. (2025). Gene editing without ex vivo culture evades genotoxicity in human hematopoietic stem cells. Cell Stem Cell *32*, 191–208.e11.
16. Frangoul, H., Locatelli, F., Bhatia, M., Mapara, M.Y., Molinari, L., Sharma, A., Lobitz, S., De Montalembert, M., Rondelli, D., Steinberg, M., et al. (2022). Efficacy and Safety of a Single Dose of Exagamglogene Autotemcel for Severe Sickle Cell Disease. Blood *140*, 29–31. https://doi.org/10.1182/blood-2022-162353.
17. Frangoul, H., Locatelli, F., Sharma, A., Bhatia, M., Mapara, M., Molinari, L., Wall, D., Liem, R.I., Telfer, P., Shah, A.J., et al. (2024). Exagamglogene Autotemcel for Severe Sickle Cell Disease. N. Engl. J. Med. *390*, 1649–1662. https://doi.org/10.1056/NEJMoa2309676.
18. Jang, Y., Feng, R., Palmer, L.E., Mayuranathan, T., Yao, Y., Mayberry, K., Zhou, S., Xu, J., Gossett, J.M., Kang, G., et al. (2025). BCL11A-deficient human erythropoiesis is impaired in vitro and after xenotransplantation into mice. Blood Adv. *9*, 2722–2732. https://doi.org/10.1182/bloodadvances.2024015574.
19. Janoudi, T., Jagdale, M., Wu, M., Gorlla, S., Zhang, P., Shao, Y., Li, L., Bowley, S.R., Marco, E., and Chang, K.-H. (2025). Nonclinical evaluation of HBG1/2 and BCL11A as genome-editing targets for the treatment of β-hemoglobinopathies. Blood Adv. *9*, 808–813. https://doi.org/10.1182/bloodadvances.2024014040.
20. Weber, L., Frati, G., Felix, T., Hardouin, G., Casini, A., Wollenschlaeger, C., Meneghini, V., Masson, C., De Cian, A., Chalumeau, A., et al. (2020). Editing a γ-globin repressor binding site restores fetal hemoglobin synthesis and corrects the sickle cell disease phenotype. Sci. Adv. *6*, eaay9392. https://doi.org/10.1126/sciadv.aay9392.
21. Frati, G., Brusson, M., Sartre, G., Mlayah, B., Felix, T., Chalumeau, A., Antoniou, P., Hardouin, G., Concordet, J.-P., Romano, O., et al. (2024). Safety and efficacy studies of CRISPR-Cas9 treatment of sickle cell disease highlights disease-specific responses. Mol. Ther. J. Am. Soc. Gene Ther. *32*, 4337–4352. https://doi.org/10.1016/j.ymthe.2024.07.015.
22. Traxler, E.A., Yao, Y., Wang, Y.-D., Woodard, K.J., Kurita, R., Nakamura, Y., Hughes, J.R., Hardison, R.C., Blobel, G.A., Li, C., and Weiss, M.J. (2016). A genome-editing strategy to treat β-hemoglobinopathies that recapitulates a mutation associated with a benign genetic condition. Nat. Med. *22*, 987–990. https://doi.org/10.1038/nm.4170.
23. Métais, J.-Y., Doerfler, P.A., Mayuranathan, T., Bauer, D.E., Fowler, S.C., Hsieh, M. M., Katta, V., Keriwala, S., Lazzarotto, C.R., Luk, K., et al. (2019). Genome editing of HBG1 and HBG2 to induce fetal hemoglobin. Blood Adv. *3*, 3379–3392. https://doi.org/10.1182/bloodadvances.2019000820.
24. Katta, V., O'Keefe, K., Li, Y., Mayuranathan, T., Lazzarotto, C.R., Wood, R.K., Levine, R.M., Powers, A., Mayberry, K., Manquen, G., et al. (2024). Development and IND-enabling studies of a novel Cas9 genome-edited autologous CD34+ cell therapy to induce fetal hemoglobin for sickle cell disease. Mol. Ther. J. Am. Soc. Gene Ther. *32*, 3433–3452. https://doi.org/10.1016/j.ymthe.2024.07.022.
25. Wimberger, S., Akrap, N., Firth, M., Brengdahl, J., Engberg, S., Schwinn, M.K., Slater, M.R., Lundin, A., Hsieh, P.-P., Li, S., et al. (2023). Simultaneous inhibition of DNA-PK and PolΘ improves integration efficiency and precision of genome editing. Nat. Commun. *14*, 4761. https://doi.org/10.1038/s41467-023-40344-4.
26. Selvaraj, S., Feist, W.N., Viel, S., Vaidyanathan, S., Dudek, A.M., Gastou, M., Rockwood, S.J., Ekman, F.K., Oseghale, A.R., Xu, L., et al. (2024). High-efficiency transgene integration by homology-directed repair in human primary cells using DNA-PKcs inhibition. Nat. Biotechnol. *42*, 731–744. https://doi.org/10.1038/s41587-023-01888-4.
27. Anzalone, A.V., Randolph, P.B., Davis, J.R., Sousa, A.A., Koblan, L.W., Levy, J.M., Chen, P.J., Wilson, C., Newby, G.A., Raguram, A., et al. (2019). Search-and-replace genome editing without double-strand breaks or donor DNA. Nature *576*, 149–157. https://doi.org/10.1038/s41586-019-1711-4.
28. Spencer, J.M., and Zhang, X. (2017). Deep mutational scanning of S. pyogenes Cas9 reveals important functional domains. Sci. Rep. *7*, 16836. https://doi.org/10.1038/s41598-017-17081-y.
29. Chen, P.J., Hussmann, J.A., Yan, J., Knipping, F., Ravisankar, P., Chen, P.-F., Chen, C., Nelson, J.W., Newby, G.A., Sahin, M., et al. (2021). Enhanced prime editing systems by manipulating cellular determinants of editing outcomes. Cell *184*, 5635–5652.e29. https://doi.org/10.1016/j.cell.2021.09.018.

30. Peterka, M., Akrap, N., Li, S., Wimberger, S., Hsieh, P.-P., Degtev, D., Bestas, B., Barr, J., van de Plassche, S., Mendoza-Garcia, P., et al. (2022). Harnessing DSB repair to promote efficient homology-dependent and -independent prime editing. Nat. Commun. *13*, 1240. https://doi.org/10.1038/s41467-022-28771-1.

31. Romano, O., and Miccio, A. (2020). GATA factor transcriptional activity: Insights from genome-wide binding profiles. IUBMB Life *72*, 10–26. https://doi.org/10.1002/iub.2169.

32. Romano, O., Petiti, L., Felix, T., Meneghini, V., Portafax, M., Antoniani, C., Amendola, M., Bicciato, S., Peano, C., and Miccio, A. (2020). GATA Factor-Mediated Gene Regulation in Human Erythropoiesis. iScience *23*, 101018. https://doi.org/10.1016/j.isci.2020.101018.

33. Behera, V., Evans, P., Face, C.J., Hamagami, N., Sankaranarayanan, L., Keller, C.A., Giardine, B., Tan, K., Hardison, R.C., Shi, J., and Blobel, G.A. (2018). Exploiting genetic variation to uncover rules of transcription factor binding and chromatin accessibility. Nat. Commun. *9*, 782. https://doi.org/10.1038/s41467-018-03082-6.

34. Chalumeau, A., Bou Dames, M., Fontana, L., Amistadi, S., Antoniou, P., Loganathan, P., Mombled, M., Corre, G., Peterka, M., Amendola, M., et al. (2025). A prime editing strategy to rewrite the γ-globin promoters and reactivate fetal hemoglobin for sickle cell disease. Blood, blood *146*, 2641–2655. https://doi.org/10.1182/blood.2024028166.

35. Antoniou, P., Dacquay, L., Mårtensson, H., Madeyski-Bengtson, K., Loyd, A.-L., Shiriaeva, A., Gordon, E., Mustfa, S., Thom, G., Hsieh, P.-P., et al. (2025). Modified pegRNAs mitigate scaffold-derived prime editing by-products. Nat. Commun. *16*, 3374. https://doi.org/10.1038/s41467-025-58653-1.

36. Antoniou, P., Hardouin, G., Martinucci, P., Frati, G., Felix, T., Chalumeau, A., Fontana, L., Martin, J., Masson, C., Brusson, M., et al. (2022). Base-editing-mediated dissection of a γ-globin cis-regulatory element for the therapeutic reactivation of fetal hemoglobin expression. Nat. Commun. *13*, 6618. https://doi.org/10.1038/s41467-022-34493-1.

37. Everette, K.A., Newby, G.A., Levine, R.M., Mayberry, K., Jang, Y., Mayuranathan, T., Nimmagadda, N., Dempsey, E., Li, Y., Bhoopalan, S.V., et al. (2023). Ex vivo prime editing of patient haematopoietic stem cells rescues sickle-cell disease phenotypes after engraftment in mice. Nat. Biomed. Eng. *7*, 616–628. https://doi.org/10.1038/s41551-023-01026-0.

38. Chen, F., Pruett-Miller, S.M., Huang, Y., Gjoka, M., Duda, K., Taunton, J., Collingwood, T.N., Frodin, M., and Davis, G.D. (2011). High-frequency genome editing using ssDNA oligonucleotides with zinc-finger nucleases. Nat. Methods *8*, 753–755. https://doi.org/10.1038/nmeth.1653.

39. Richardson, C.D., Ray, G.J., DeWitt, M.A., Curie, G.L., and Corn, J.E. (2016). Enhancing homology-directed genome editing by catalytically active and inactive CRISPR-Cas9 using asymmetric donor DNA. Nat. Biotechnol. *34*, 339–344. https://doi.org/10.1038/nbt.3481.

40. Locatelli, F., Lang, P., Wall, D., Meisel, R., Corbacioglu, S., Li, A.M., de la Fuente, J., Shah, A.J., Carpenter, B., Kwiatkowski, J.L., et al. (2024). Exagamglogene Autotemcel for Transfusion-Dependent β-Thalassemia. N. Engl. J. Med. *390*, 1663–1676. https://doi.org/10.1056/NEJMoa2309673.

41. Dacquay, L.C., Antoniou, P., Mentani, A., Selfjord, N., Mårtensson, H., Hsieh, P.P., Mustfa, S., Thom, G., Wimberger, S., Firth, M., et al. (2025). Dual inhibition of DNA-PK and PolΘ boosts precision of diverse prime editing systems. Nat. Commun *16*, 4290. https://doi.org/10.1038/s41467-025-59708-z.

42. Schimmel, J., Muñoz-Subirana, N., Kool, H., van Schendel, R., van der Vlies, S., Kamp, J.A., de Vrij, F.M.S., Kushner, S.A., Smith, G.C.M., Boulton, S.J., et al. (2023). Modulating mutational outcomes and improving precise gene editing at CRISPR-Cas9-induced breaks by chemical inhibition of end-joining pathways. Cell Rep. *42*, 112019. https://doi.org/10.1016/j.celrep.2023.112019.

43. Riesenberg, S., Kanis, P., Macak, D., Wollny, D., Düsterhöft, D., Kowalewski, J., Helmbrecht, N., Maricic, T., and Pääbo, S. (2023). Efficient high-precision homology-directed repair-dependent genome editing by HDRobust. Nat. Methods *20*, 1388–1399. https://doi.org/10.1038/s41592-023-01949-1.

44. Koblan, L.W., Doman, J.L., Wilson, C., Levy, J.M., Tay, T., Newby, G.A., Maianti, J. P., Raguram, A., and Liu, D.R. (2018). Improving cytidine and adenine base editors by expression optimization and ancestral reconstruction. Nat. Biotechnol. *36*, 843–846. https://doi.org/10.1038/nbt.4172.

45. Hanna, R., Frangoul, H., McKinney, C., Pineiro, L., Mapara, M., Dalal, J., Chang, K.-H., Jaskolka, M., Kim, K., Farrington, D.L., et al. (2023). AsCas12a Gene Editing of *HBG1/2* Promoters with EDIT-301 Results in Rapid and Sustained Normalization of Hemoglobin and Increased Fetal Hemoglobin in Patients with Severe Sickle Cell Disease and Transfusion-Dependent Beta-Thalassemia. Blood *142*, 4996. https://doi.org/10.1182/blood-2023-187397.

46. De Dreuzy, E., Heath, J., Zuris, J.A., Sousa, P., Viswanathan, R., Scott, S., Da Silva, J., Ta, T., Capehart, S., Wang, T., et al. (2019). EDIT-301: An Experimental Autologous Cell Therapy Comprising Cas12a-RNP Modified mPB-CD34+ Cells for the Potential Treatment of SCD. Blood *134*, 4636. https://doi.org/10.1182/blood-2019-130256.

47. Gupta, A.O., Sharma, A., Frangoul, H., Kanter, J., Mapara, M.Y., Dalal, J., Alavi, A., Jaroscak, J.J., Ayala, E., DiPersio, J.F., et al. (2026). Base Editing of HBG1 and HBG2 Promoters for Sickle Cell Disease. N. Engl. J. Med. *394*, 1824–1835. https://doi.org/10.1056/NEJMoa2504835.

48. Lai, Y., Liu, R., Wang, L., Ma, X.-K., Li, Y., Yang, G., Shi, L., Guo, Y.-L., Wei, Z., Zhou, X., et al. (2026). Clinical application of base editing for treating β-thalassaemia. Nature *653*, 923–932. https://doi.org/10.1038/s41586-026-10342-9.

49. Hanna, R., Frangoul, H., Pineiro, L., McKinney, C., Mapara, M., Dalal, J., Rangarajan, H.G., Atkins, H., Sharma, A., Chang, K.-H., et al. (2026). CRISPR-Cas12a Gene Editing of HBG1 and HBG2 Promoters to Treat Sickle Cell Disease. N. Engl. J. Med. *394*, 1281–1291. https://doi.org/10.1056/NEJMoa2415550.

50. Han, W., Qiu, H.-Y., Sun, S., Fu, Z.-C., Wang, G.-Q., Qian, X., Wang, L., Zhai, X., Wei, J., Wang, Y., et al. (2023). Base editing of the HBG promoter induces potent fetal hemoglobin expression with no detectable off-target mutations in human HSCs. Cell Stem Cell *30*, 1624–1639.e8. https://doi.org/10.1016/j.stem.2023.10.007.

51. Magnani, A., Pondarré, C., Bouazza, N., Magalon, J., Miccio, A., Six, E., Roudaut, C., Arnaud, C., Kamdem, A., Touzot, F., et al. (2019). Extensive multilineage analysis in patients with mixed chimerism after allogeneic transplantation for sickle cell disease: insight into hematopoiesis and engraftment thresholds for gene therapy. Haematologica *105*, 1240–1247. https://doi.org/10.3324/haematol.2019.227561.

52. Lian, X., Chatterjee, S., Sun, Y., Dilliard, S.A., Moore, S., Xiao, Y., Bian, X., Yamada, K., Sung, Y.-C., Levine, R.M., et al. (2024). Bone-marrow-homing lipid nanoparticles for genome editing in diseased and malignant haematopoietic stem cells. Nat. Nanotechnol. *19*, 1409–1417. https://doi.org/10.1038/s41565-024-01680-8.

53. Brinkman, E.K., Chen, T., De Haas, M., Holland, H.A., Akhtar, W., and Van Steensel, B. (2018). Kinetics and Fidelity of the Repair of Cas9-Induced Double-Strand DNA Breaks. Mol. Cell *70*, 801–813.e6. https://doi.org/10.1016/j.molcel.2018.04.016.

54. Collins, A.R., Ma, A.G., and Duthie, S.J. (1995). The kinetics of repair of oxidative DNA damage (strand breaks and oxidised pyrimidines) in human cells. Mutat. Res. *336*, 69–77. https://doi.org/10.1016/0921-8777(94)00043-6.

55. Wang, J., Sadeghi, C.A., and Frock, R.L. (2024). DNA-PKcs suppresses illegitimate chromosome rearrangements. Nucleic Acids Res. *52*, 5048–5066. https://doi.org/10.1093/nar/gkae140.

56. Yan, J., Oyler-Castrillo, P., Ravisankar, P., Ward, C.C., Levesque, S., Jing, Y., Simpson, D., Zhao, A., Li, H., Yan, W., et al. (2024). Improving prime editing with an endogenous small RNA-binding protein. Nature *628*, 639–647. https://doi.org/10.1038/s41586-024-07259-6.

57. Anzalone, A.V., Gao, X.D., Podracky, C.J., Nelson, A.T., Koblan, L.W., Raguram, A., Levy, J.M., Mercer, J.A.M., and Liu, D.R. (2021). Programmable deletion, replacement, integration and inversion of large DNA sequences with twin prime editing. Nat. Biotechnol. *40*, 731–740. https://doi.org/10.1038/s41587-021-01133-w.

58. Sousa, A.A., Hemez, C., Lei, L., Traore, S., Kulhankova, K., Newby, G.A., Doman, J. L., Oye, K., Pandey, S., Karp, P.H., et al. (2024). Systematic optimization of prime editing for the efficient functional correction of CFTR F508del in human airway epithelial cells. Nat. Biomed. Eng. *9*, 7–21. https://doi.org/10.1038/s41551-024-01233-3.

59. Yap, T.A., LoRusso, P., Miller, R.E., Kristeleit, R., Paulovich, A.G., McMorn, S., Oplustil O'Connor, L., Lombardi, B., Marco-Casanova, P., Gangl, E.T., et al. (2025). The DNA-PK inhibitor AZD7648 alone or combined with pegylated liposomal doxorubicin in patients with advanced cancer: results of a first-in-human

Phase I/IIa study. Br. J. Cancer *133*, 168–177. https://doi.org/10.1038/s41416-025-03053-x.

60. Cullot, G., Aird, E.J., Schlapansky, M.F., Yeh, C.D., van de Venn, L., Vykhlyantseva, I., Kreutzer, S., Mailänder, D., Lewków, B., Klermund, J., et al. (2025). Genome editing with the HDR-enhancing DNA-PKcs inhibitor AZD7648 causes large-scale genomic alterations. Nat. Biotechnol. *43*, 1778–1782. https://doi.org/10.1038/s41587-024-02488-6.

61. White, N., Hu, Y.-T., Chalk, J.A., Kurgan, G., Naseem, A., Schmaljohn, E., Sturgeon, M., Cavazza, A., Thrasher, A.J., and Turchiano, G. (2026). DNA-PKcs inhibitor AZD7648 reveals sgRNA cross-contaminants and enhanced sensitivity of genome engineering off-target activity in HSPCs. Nucleic Acids Res. *54*, gkag318. https://doi.org/10.1093/nar/gkag318.

62. Studier, F.W. (2005). Protein production by auto-induction in high density shaking cultures. Protein Expr. Purif. *41*, 207–234. https://doi.org/10.1016/j.pep.2005.01.016.

63. Brusson, M., Chalumeau, A., Martinucci, P., Romano, O., Felix, T., Poletti, V., Scaramuzza, S., Ramadier, S., Masson, C., Ferrari, G., et al. (2023). Novel lentiviral vectors for gene therapy of sickle cell disease combining gene addition and gene silencing strategies. Mol. Ther. Nucleic Acids *32*, 229–246. https://doi.org/10.1016/j.omtn.2023.03.012.

OMTN, Volume *37*

Supplemental information

Enhanced γ-globin reactivation and sickle cell correction through a repressor-to-activator motif switch in the *HBG1/2* promoters

Anne Chalumeau, Panagiotis Antoniou, Maria Bou Dames, Pierre Martinucci, Elena Retana, Pragya Gupta, Mike Firth, Muralidhar Reddivari, Aathira Sarath Chandran, Jonathan S. Yen, Martin Peterka, Marcello Maresca, Mégane Brusson, and Annarita Miccio

Table S1: List of pegRNAs and springRNAs sequences used in the study (Provided separately in Excel format)

Table S2: List of ssODNs sequences targeting the -115 region of the *HBG* promoters

ssODNs name	spacer 5'-3'
sg115_D1(+)	CCCTGGCTAAACTCCACCCATGGGTTGGCCAGCCTTGCCTCTGCTGGGGACCCGATAACCAATAGCCTTGACAAGGCAAACTTGACCAATAGTCTTA
sg115_D1 (-)	TAAGACTATTGGTCAAGTTTGCCTTGTCAAGGCTATTGGTTATCGGGTCCCCAGCAGAGGCAAGGCTGGCCAACCCATGGGTGGAGTTTAGCCAGGG
sg115_D2 (+)	CTGAAACGGTCCCTGGCTAAACTCCACCCATGGGTTGGCCAGCCTTGCCTCTGCTGGGGACCCGATAACCAATAGCCTTGACAAGGCAAACTTGACCAATAGTCTTAGAGTATCCAG
sg115_D2 (-)	CTGGATACTCTAAGACTATTGGTCAAGTTTGCCTTGTCAAGGCTATTGGTTATCGGGTCCCCAGCAGAGGCAAGGCTGGCCAACCCATGGGTGGAGTTTAGCCAGGGACCGTTTCAG
sg115_D3 (+)	AAATATCTGTCTGAAACGGTCCCTGGCTAAACTCCACCCATGGGTTGGCCAGCCTTGCCTCTGCTGGGGACCCGATAACCAATAGCCTTGACAAGGCAAACTTGACCAATAGTCTTAGAGTATCCAGTGAGGCCAGG
sg115_D3 (-)	CCTGGCCTCACTGGATACTCTAAGACTATTGGTCAAGTTTGCCTTGTCAAGGCTATTGGTTATCGGGTCCCCAGCAGAGGCAAGGCTGGCCAACCCATGGGTGGAGTTTAGCCAGGGACCGTTTCAGACAGATATTT
sg115_D4 (+)	AAATATCTGTCTGAAACGGTCCCTGGCTAAACTCCACCCATGGGTTGGCCAGCCTTGCCTCTGCTGGGGACCCGATAACCAATAGCCTTGACAAGGCAAACTTGACCAATAGTCTTAGAGTATCCAGTGAGGCCAGGGGCCGGCGGCTGGCTAGGGATGAAGAATAAAAGGA AGCACCCTTCAGCAG
sg115_D4 (-)	CTGCTGAAGGGTGCTTCCTTTTATTCTTCATCCCTAGCCAGCCGCCGGCCCCTGGCCTCACTGGATACTCTAAGACTATTGGTCAAGTTTGCCTTGTCAAGGCTATTGGTTATCGGGTCCCCAGCAGAGGCAAGGCTGGCCAACCCATGGGTGGAGTTTAGCCAGGGACCGT
sg115_D5 (+)	TTAAGCAGCAGTATCCTCTTGGGGGCCCCTTCCCCACACTATCTCAATGCAAATATCTGTCTGAAACGGTCCCTGGCTAAACTCCACCCATGGGTTGGCCAGCCTTGCCTCTGCTGGGGACCCGATAACCAATAGCCTTGACAAGGCAAACTTGACCAATAGTCTTAGAGTA TCCAGTGAGGCCAGG
sg115_D5 (-)	CCTGGCCTCACTGGATACTCTAAGACTATTGGTCAAGTTTGCCTTGTCAAGGCTATTGGTTATCGGGTCCCCAGCAGAGGCAAGGCTGGCCAACCCATGGGTGGAGTTTAGCCAGGGACCGTTTCAGACAGATATTTGCATTGAGATAGTGTGGGGAAGGGGCCCCCAAGAG

ssODNs are labeled numerically (1–5) to denote distinct designs, with (+) and (–) indicating the DNA strand targeted by the ssODN.

Table S3: List of primers used for on- and off-target analysis by deep sequencing

Deep sequencing for PEn experiments				
**Analysis**	**Amplified region**	**F/R**	**N.**	**Sequence (5' to 3')**
On-target	*chr11_5271202/5276126*	F	1	TCGTCGGCAGCGTCAGATGTGTATAAGAGACAGAAGCGGAATGACTGAATCGGAACAAGG
			2	TCGTCGGCAGCGTCAGATGTGTATAAGAGACAGCGTTGGAATGACTGAATCGGAACAAGG
			3	TCGTCGGCAGCGTCAGATGTGTATAAGAGACAGGTCAGGAATGACTGAATCGGAACAAGG
			4	TCGTCGGCAGCGTCAGATGTGTATAAGAGACAGACAGGGAATGACTGAATCGGAACAAGG
			5	TCGTCGGCAGCGTCAGATGTGTATAAGAGACAGCAGTGGAATGACTGAATCGGAACAAGG
			6	TCGTCGGCAGCGTCAGATGTGTATAAGAGACAGTGTCGGAATGACTGAATCGGAACAAGG
		R	1	GTCTCGTGGGCTCGGAGATGTGTATAAGAGACAGAGCAGCACTGGCCTCACTGGATACTCT
			2	GTCTCGTGGGCTCGGAGATGTGTATAAGAGACAGCCTTGCACTGGCCTCACTGGATACTCT
			3	GTCTCGTGGGCTCGGAGATGTGTATAAGAGACAGGAACGCACTGGCCTCACTGGATACTCT
			4	GTCTCGTGGGCTCGGAGATGTGTATAAGAGACAGTTGGGCACTGGCCTCACTGGATACTCT
			5	GTCTCGTGGGCTCGGAGATGTGTATAAGAGACAGACAGGCACTGGCCTCACTGGATACTCT
			6	GTCTCGTGGGCTCGGAGATGTGTATAAGAGACAGCAGTGCACTGGCCTCACTGGATACTCT
			7	GTCTCGTGGGCTCGGAGATGTGTATAAGAGACAGCTACGCACTGGCCTCACTGGATACTCT
			8	GTCTCGTGGGCTCGGAGATGTGTATAAGAGACAGGATGGCACTGGCCTCACTGGATACTCT
			9	GTCTCGTGGGCTCGGAGATGTGTATAAGAGACAGGGATGCACTGGCCTCACTGGATACTCT
			10	GTCTCGTGGGCTCGGAGATGTGTATAAGAGACAGTGTCGCACTGGCCTCACTGGATACTCT
Off-target	*chr2_176540234*	F	1	ATTGGGGGAGACAGACCCATG
		R	2	CATTTTTATCCAGCTTGGCAGGGG

F, forward primers; R, reverse primers

Table S4: Crispresso2 analysis (Provided separately in Excel format)

Table S5: List of primers used for RT-qPCR analysis

Amplified region	F/R	Sequence (5' to 3')
HBA	F	CGGTCAACTTCAAGCTCCTAA
	R	ACAGAAGCCAGGAACTTGTC
HBB	F	GCAAGGTGAACGTGGATGAAGT
	R	TAACAGCATCAGGAGTGGACAGA
HBG1 + HBG2	F	CCTGTCCTCTGCCTCTGCC
	R	GGATTGCCAAAACGGTCAC
CDKN1A	F	CAGCATGACAGATTTCTACCACTC
	R	CTCGCGCTTCCAGGACTG
GAPDH	F	ACCACAGTCCATGCCATCACT
	R	CCATCACGCCACAGTTTCC

Table S6: Primers and probe used for the 4.9-kb deletion ddPCR analysis

**ddPCR analysis**			
	F/R	Sequence (5' to 3')	Fluorophore
primers	F	AAAGAGAGGTGGAAATGAGG	
	R	CCACTTTGACTGAGCCAATA	
HBG1	probe	CCAGTAGAAAGAACTTTCATCTTTCCCTC	FAM
HBG2	probe	CTTTCCCCTATTTTTGTTATTCGTTTTA	VIC

Figure S1

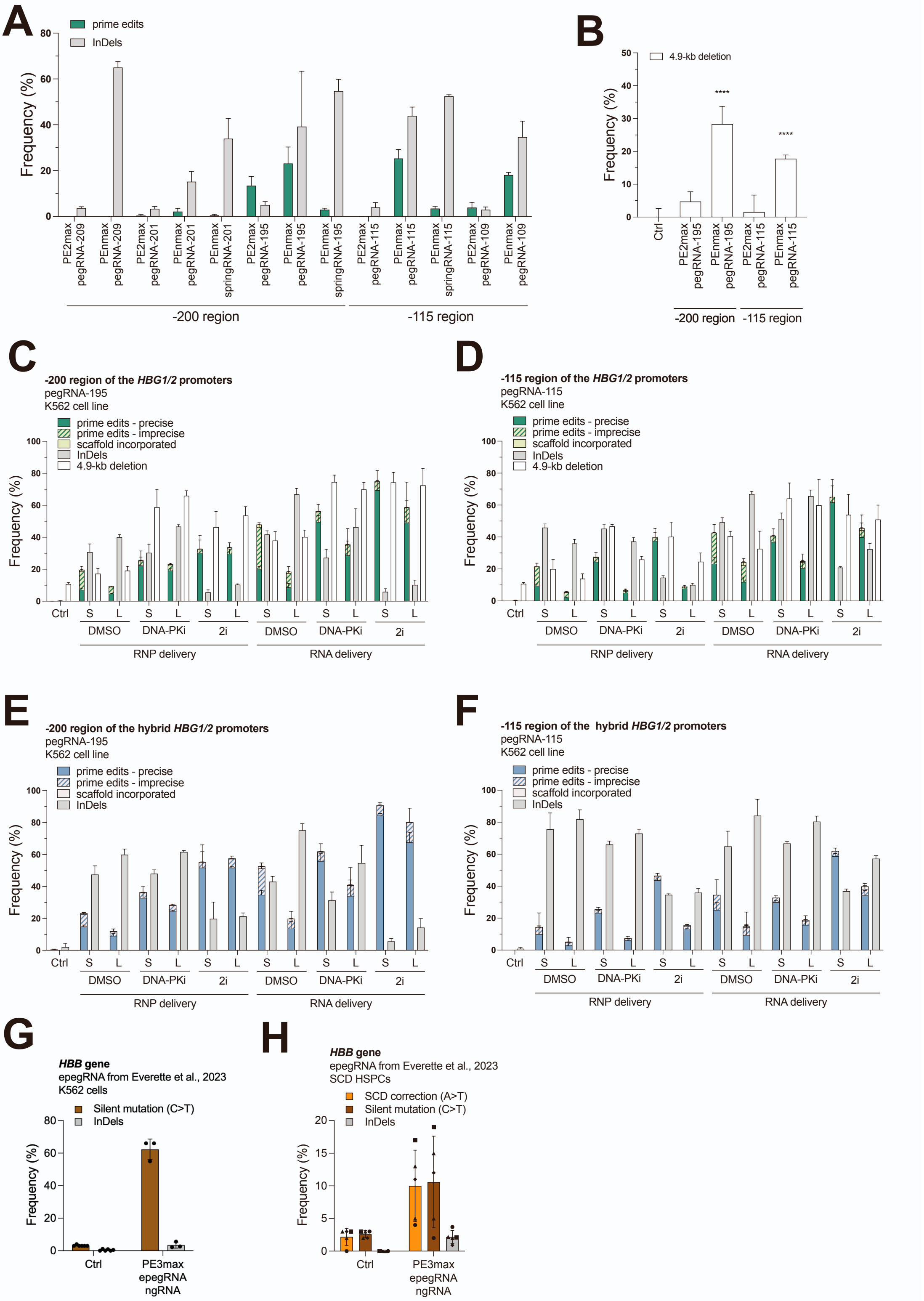

Figure S1: Design and screening of pegRNAs and springRNAs targeting the *HBG1/2* promoters in K562 cells.

(A) Frequency of the desired insertion (prime edits) and InDels in the *HBG1/2* promoters induced by pegRNAs or the springRNAs in combination with PEnmax or PE2max in K562 cells. The *HBG1/2* promoters were amplified and subjected to Sanger sequencing. Data were analyzed using the TIDE software. Bars represent the mean ± SD of 3 biologically independent replicates.
(B) The frequency of the 4.9-kb deletion was measured by ddPCR in control (Ctrl, cells transfected with Tris-EDTA, TE) and edited cells.
(C, D) Percentage of total *HBG1/2* promoters containing the total prime edits (precise, imprecise, scaffold incorporated) and the InDels induced by (C) pegRNA-195_S or pegRNA-115_L, or (D) pegRNA-115_S or pegRNA-115_L, delivered as RNA or RNP in K562 cells treated or not with DNA-PKi or DNA-PKi and Polθi inhibitors. The *HBG1/2* promoters were amplified and subjected to NGS. Data were analyzed with the CRISPResso2 webtool. We report also the frequency of the 4.9-kb deletion measured by ddPCR for control (ctrl, TE-transfected cells) and prime-edited conditions. Bars represent the mean ± SD of 3 technical replicates.
(E, F) Percentage of the hybrid *HBG1/2* promoters containing the prime edits (precise, imprecise, scaffold incorporated) and InDels induced by (E) pegRNA-195_S or pegRNA-115_L or (F) pegRNA-115_S or pegRNA-115_L, delivered as RNA or RNP in K562 cells treated or not with DNA-PKi or DNA-PKi and Polθi inhibitors. The hybrid *HBG1/2* promoters were amplified and subjected to NGS. Data were analyzed with the CRISPResso2 webtool. Bars represent the mean ± SD of 3 biological replicates.
(G) Frequency of insertion of the silent mutation in the *HBB* gene, and InDels in the *HBB* gene induced by epegRNA PE3max in K562 cells (n=3). The *HBB* gene was amplified and subjected to Sanger sequencing. Data were analyzed using the TIDE software. Bars represent the mean ± SD of 3 biologically independent replicates.
(H) Frequency of SCD mutation correction and insertion of a silent mutation, and InDels in the *HBB* gene induced by epegRNA PE3max in HSPCs from patients with SCD (n=4 donors). The *HBB* gene was amplified and subjected to Sanger sequencing. Data were analyzed using the TIDE software. Bars represent the mean ± SD of 5 biologically independent replicates.

Figure S2

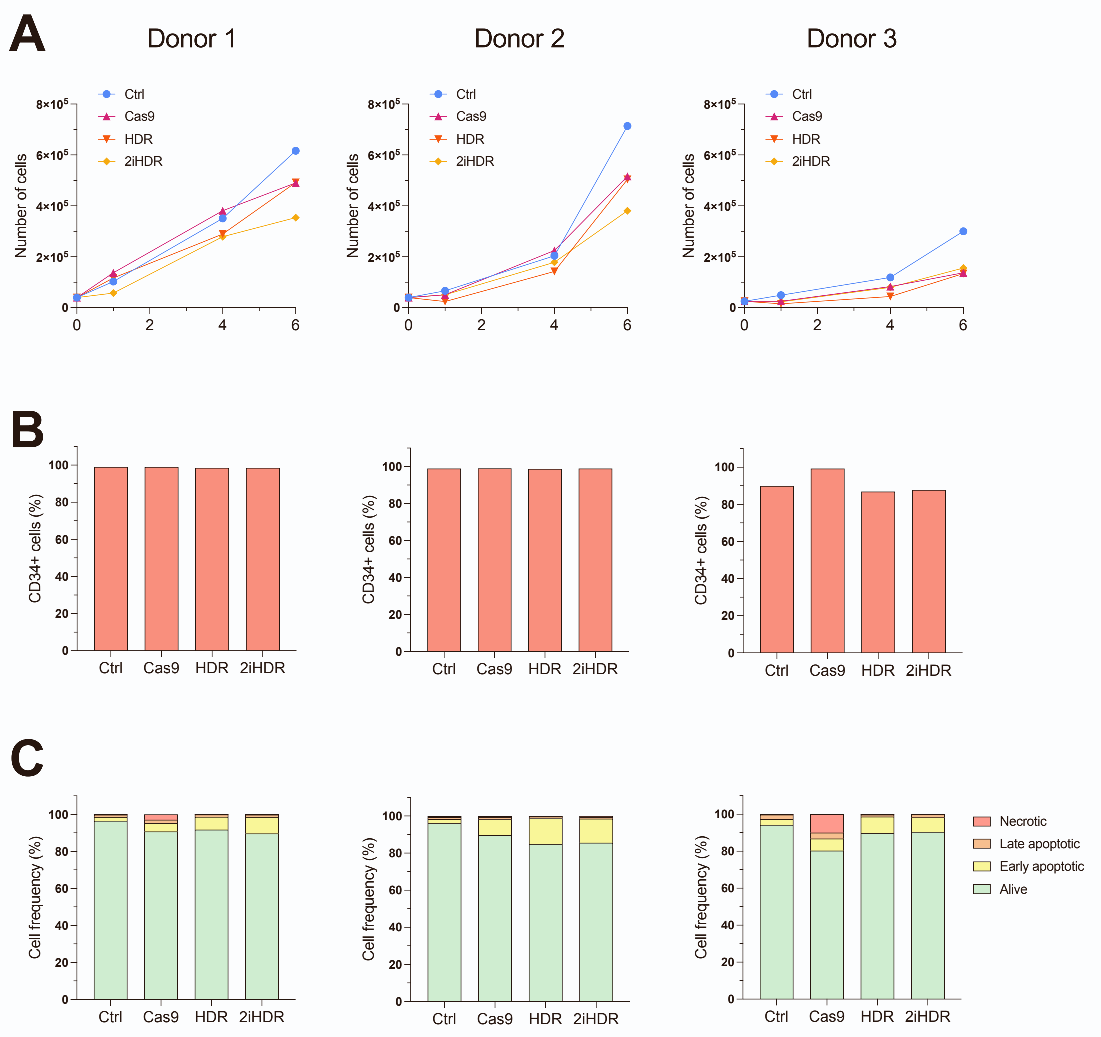

Figure S2: Cell recovery, viability, and DNA damage response in control and edited HSPCs

(A) Number of HSPCs 1, 2, 4 and 6 days post transfection in control (TE-transfected) and edited samples.

(B) Frequency of CD34+ HSPCs 24 h post transfection in control (TE-transfected) and edited samples, as measured by flow cytometry.

(C) Frequency of alive, early apoptotic, late apoptotic, and necrotic HSPCs 24 h post transfection, as measured by flow cytometry after annexin-V staining in control (mock transfected) and edited samples.

(A-C) Data are shown for 3 different SCD donors, including one bone marrow sample (donor 3).

Figure S3

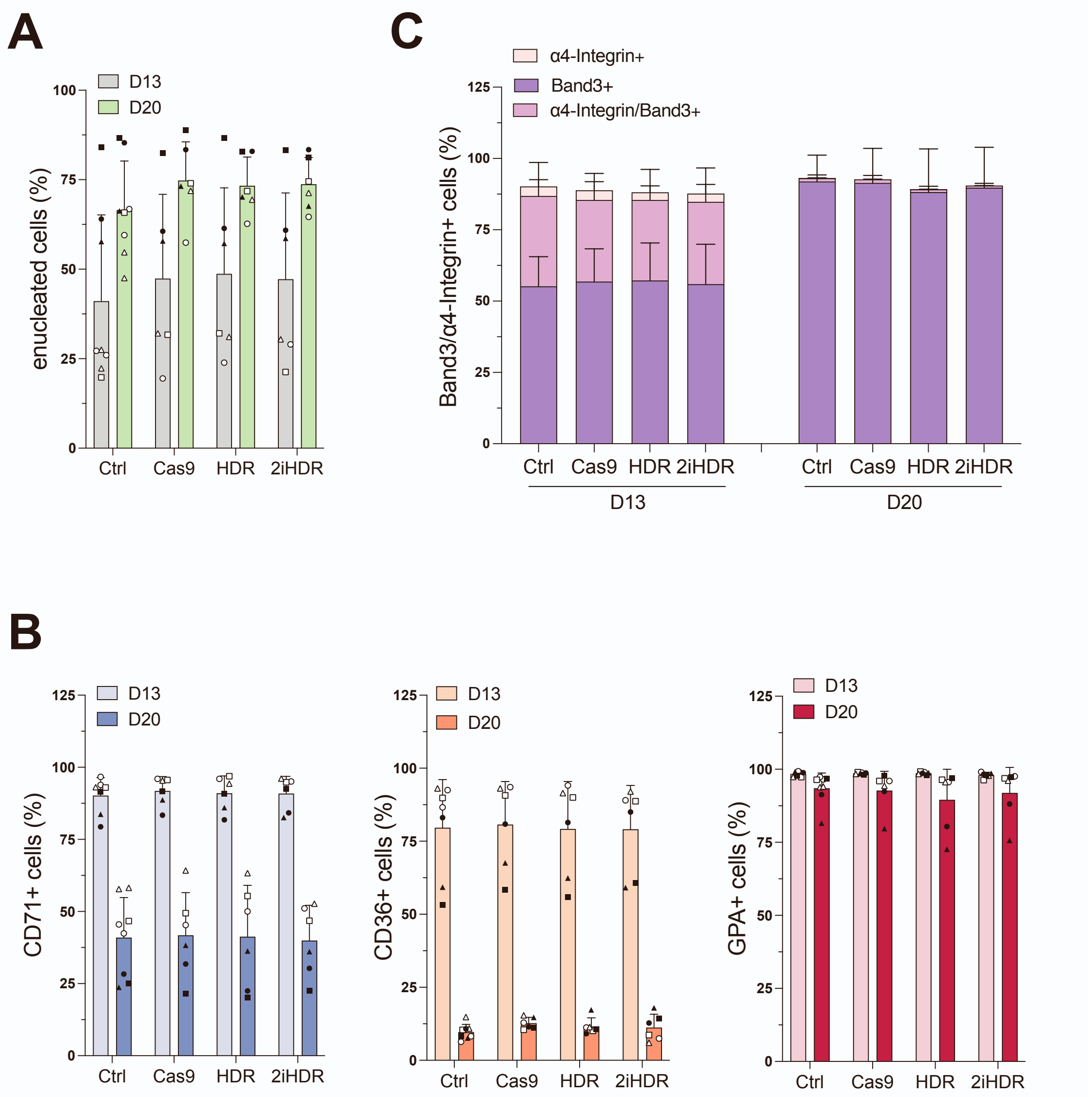

Figure S3: The Cas9-based strategies do not impact the erythroid differentiation
(A) Frequency of enucleated cells at day 13 and 20 of erythroid differentiation, as measured by flow cytometry analysis after DRAQ5 nuclear staining in control and edited samples. Data are expressed as mean ± SD (n=6 biologically independent experiments; 5 different donors). No statistical differences were observed between control and edited samples using two-way ANOVA with Tukey's multiple comparisons test.
(B) Frequency of (left) CD71+, (middle) CD36+ and (right) GPA+ cells at day 13 and 20 of the erythroid differentiation, as measured by flow cytometry analysis of CD36, CD71, and GPA erythroid markers in control and edited samples. Data are expressed as mean ± SD (n=6 biologically independent experiments; 5 different donors). No statistical differences were observed between control and edited samples using two-way ANOVA with Tukey's multiple comparisons test.

(C) Frequency of α4-Integrin+, Band3+ and α4-Integrin+/Band3+ in 7AAD-/GPA+ cells at day 13 and 20 of erythroid differentiation, as measured by flow cytometry analysis of α4-Integrin and Band3 erythroid markers in control and edited samples. Data are expressed as mean ± SD (n=6 biologically independent experiments; 5 different donors). No statistical differences were observed between control and edited samples using two-way ANOVA with Tukey's multiple comparisons test.

Figures S4

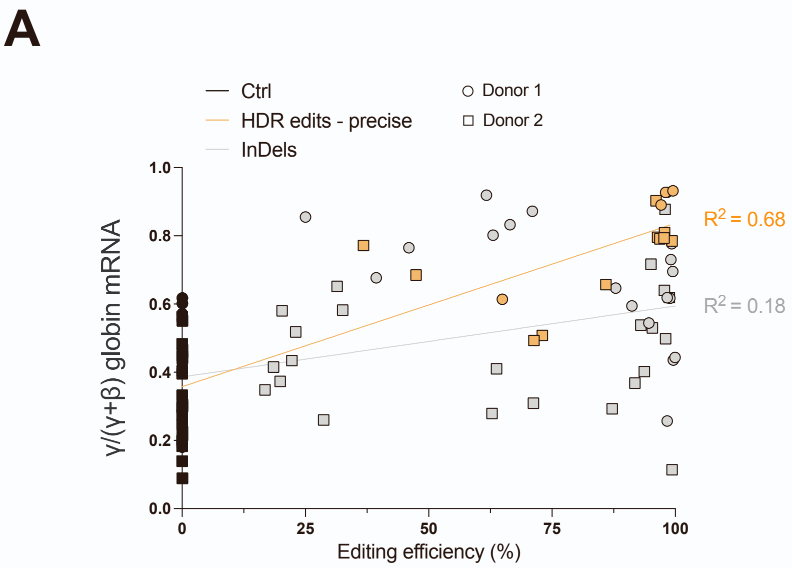

Figure S4: Cas9-HDR-edited BFU-E express high HbF levels.
(A) Correlation between γ-globin mRNA expression and editing efficiency in single erythroid BFU-E (n=32 to 74 single BFU-E for each group). γ-globin mRNA expression was normalized to α-globin mRNA and expressed as a percentage of the total β- and γ- globin mRNA. For BFU-E containing the HDR precise edits, R^2 = 0.68, Y = 0,004792*X + 0,3577; for BFU-E containing InDels, R^2 = 0.18, Y = 0,002081*X + 0,3862 (simple linear regression).
